# 1*H*-Pyrazolo[3,4-*b*]quinolines: Synthesis and Properties over 100 Years of Research

**DOI:** 10.3390/molecules27092775

**Published:** 2022-04-26

**Authors:** Andrzej Danel, Ewa Gondek, Mateusz Kucharek, Paweł Szlachcic, Arkadiusz Gut

**Affiliations:** 1 Faculty of Materials Engineering and Physics, Cracow University of Technology, Podchorążych Str. 1, 30-084 Krakow, Poland; egondek@pk.edu.pl; 2 Faculty of Food Technology, University of Agriculture in Krakow, Balicka Str. 122, 30-149 Krakow, Poland; mateusz.kucharek@urk.edu.pl (M.K.); pawel.szlachcic@urk.edu.pl (P.S.); 3 Faculty of Chemistry, Jagiellonian University, Gronostajowa Str. 2, 30-387 Krakow, Poland; arkadiusz.gut@uj.edu.pl

**Keywords:** biological properties, fluorescence, fluorescent sensors, Friedländer condensation, multicomponent reaction, 1*H*-pyrazolo[3,4-*b*]quinolines

## Abstract

This paper summarises a little over 100 years of research on the synthesis and the photophysical and biological properties of 1*H*-pyrazolo[3,4-*b*]quinolines that was published in the years 1911–2021. The main methods of synthesis are described, which include Friedländer condensation, synthesis from anthranilic acid derivatives, multicomponent synthesis and others. The use of this class of compounds as potential fluorescent sensors and biologically active compounds is shown. This review intends to summarize the abovementioned aspects of 1*H*-pyrazolo[3,4-*b*]quinoline chemistry. Some of the results that are presented in this publication come from the laboratories of the authors of this review.

## 1. Introduction

**Figure 1:**
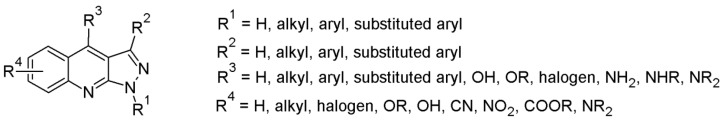
The structure of 1*H*-pyrazolo[3,4-*b*]quinoline.

1*H*-Pyrazolo[3,4-*b*]quinolines are three-membered azaheterocyclic systems that are composed of a pyrazole-and-quinoline fragment (Figure 1). The parent structure can be modified with a number of substituents that have a great influence on the physical, photophysical and biological properties.

**Figure 2:**
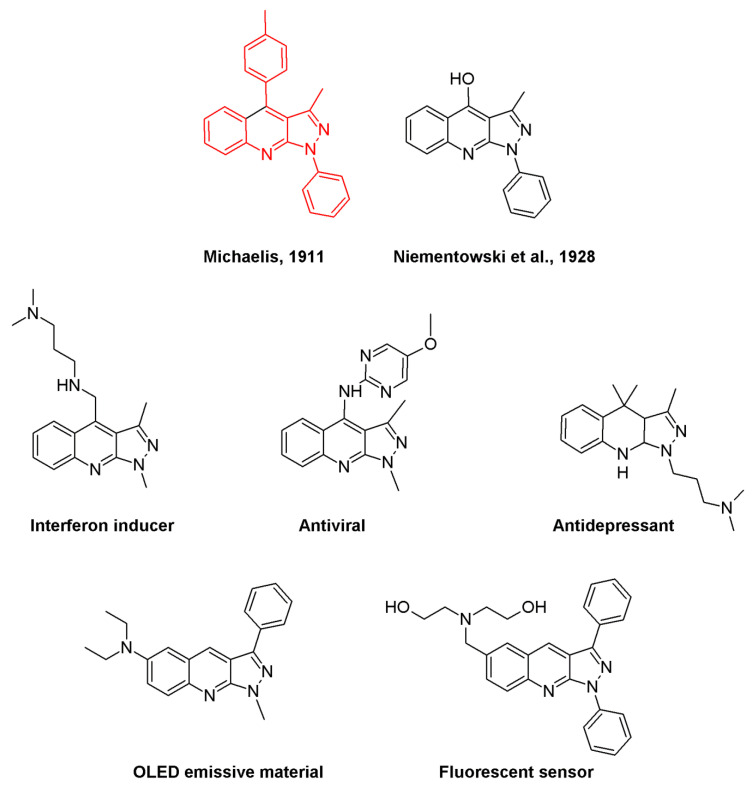
Various 1*H*-pyrazolo[3,4-*b*]quinoline structures.

The first synthesis of this class of compounds was described in 1911 by Michaelis; however, the author incorrectly presented their structures. He concluded that the obtained compounds were a benzylidene derivative of 5-*N*-phenylamino-3-methyl-1-phenylpyrazole. This structure is marked in red in Figure 2 [1]. His results were later verified by other researchers. For this reason, the discoverer of this class of compounds should be considered as Niementowski and colleagues, who in their work presented the first structure of 1*H*-pyrazolo[3,4-*b*]quinoline in 1928 [2]. In the interwar period, there were some works by Koćwa, who also synthesised this system [3,4]. The remaining works on the synthesis of these compounds were published after 1945.

Michaelis noticed that the compounds that were obtained by him were characterised by intense fluorescence, and, indeed, a significant part of pyrazolo[3,4-*b*]quinolines exhibits emission properties, both in solutions and even in a solid state. These properties have been used in the synthesis of fluorescent sensors for various cations [5]. In addition, pyrazolo[3,4-*b*]quinolines were tested as emission materials in organic electroluminescent cells (OLEDs). The summary of this research was published some time ago in our review article [6]. It is also necessary to mention the biological properties of this class of compounds, which were largely studied in the first stage of 1*H*-pyrazolo[3,4-*b*]quinoline development, and it is now observed that more and more research groups are interested in these heterocycles. This aspect will be discussed later in this review (Figure 2).

## 2. The Main Synthesis Method of 1*H*-Pyrazolo[3,4-*b*]quinoline

**Figure 3:**
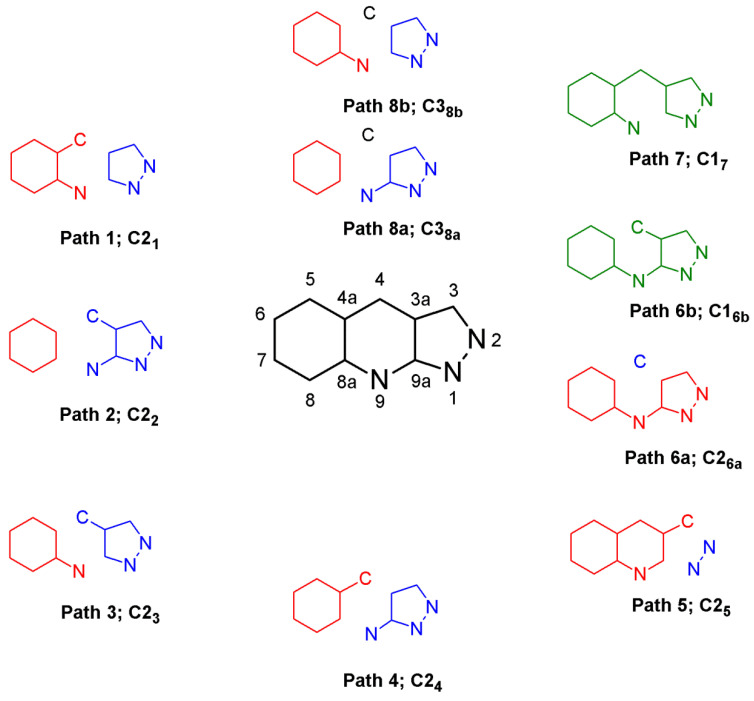
Various methods for constructing the 1*H*-pyrazolo[3,4-*b*]quinoline carbon skeleton.

The following scheme for the synthesis of pyrazoloquinolines is based on the methodology for the synthesis of the quinoline system, which is described in detail in one of the Houben–Weil volumes on quinoline synthesis [7] (Figure 3). The first and oldest method of pyrazoloquinolines synthesis is a two-component reaction (C2_1_), in which the Friedländer condensation of anthranilaldehyde, *o*-aminoacetophenones and *o*-aminobenzophenones, and the appropriate pyrazolones, are used (Figure 3; Path 1). As a result of the reaction, a bond is formed between the N9 nitrogen and the C9a carbon, and between the C3a and C4 carbon atoms. The reaction is limited by the availability of *o*-aminocarbonyl compounds. The synthesis of the Niementowski and Pfitzinger quinolines can also be used here, although to a lesser extent than in the first case.

The second path is essentially the reverse of the first methodology, where the *o*-aminocarbonyl system is linked to the pyrazole moiety. In a two-component reaction (C2_2_), a bond is formed between the C8a carbon and the N9 nitrogen, and between the carbons C4a and C4 (Figure 3; Path 2).

The two-component reaction (C2_3_) in which one of the reactants is an aromatic amine and the other is a pyrazole derivative is one of the most valuable syntheses of the pyrazoloquinoline system. The entire spectrum of substituted anilines is commercially available, while the synthesis of pyrazole derivatives that contain an aldehyde or ketone group is not difficult. In this reaction, a bond is formed between C4 and C4a carbon, and between nitrogen N9 and C9a carbon (Figure 3; Path 3).

The reaction of 5-aminopyrazoles and the *o*-halogen derivatives of aromatic carboxylic acids has some importance in the synthesis of pyrazoloquinolines that are substituted at the 4-chlorine/bromine or hydroxyl position. In this two-component reaction (C2_4_), a bond is formed between the nitrogen atom N9 and the carbon C8a, and between the carbon carbon and the carbon C3a (Figure 3; Path 4).

After studying the literature on the synthesis of pyrazoloquinolines, it seems that Path 5 is one of the most exploited procedures for synthesising this system. It uses quinoline derivatives (aldehydes, nitriles) and the appropriate hydrazines. The resulting derivatives are later further modified, and most often in terms of the synthesis of biologically active compounds. In this two-component reaction (C2_5_), a bond is formed between the C9a carbon and the N1 nitrogen, and between the N2 nitrogen and the C3 carbon (Figure 3; Path 5).

Another synthetic procedure of pyrazoloquinolines is a two-component reaction (C2_6a_) and a one-component reaction (C1_6b_). Both routes are based on 5-*N*-arylpyrazole derivatives. In the first case, cyclization takes place with the use of an aromatic aldehyde, and, in the second case, an ester group is used, which is attached to the pyrazole ring in position 4. In the first situation, bonds between the C4 and C4a/C3a carbons are formed. In the second case, bonds between C4 and C4a are formed. The ring-closure is also performed by using the Vilsmeier–Haack formylation reaction (Figure 3; Path 6).

The use of a one-component system (C1_7_), where the reductive cyclization of the *o*-nitrobenzylidene system is used, is practically irrelevant when it comes to the synthesis of pyrazoloquinolines. A bond is formed between the nitrogen N9 and the C9a carbon in the pyrazole ring (Figure 3; Path 7).

Finally, we should mention multicomponent reactions, which are very popular in the synthesis of heterocyclic systems. This method is suitable for both the synthesis of the fully aromatic pyrazoloquinoline system, or with the hydrogenated moiety in either the middle ring or the carbocyclic system. The N9-nitrogen-contributing system may be an amine pyrazole or an aromatic amine (C3_8a_ or C3_8b_). The C4-bound fragment is most often derived from an aromatic or an aliphatic aldehyde (Figure 3; Path 8).

### 2.1. The Friedländer Synthesis Based on o-Aminoaldehydes, o-Aminoacetophenones and o-Aminobenzophenones

**Figure 4:**
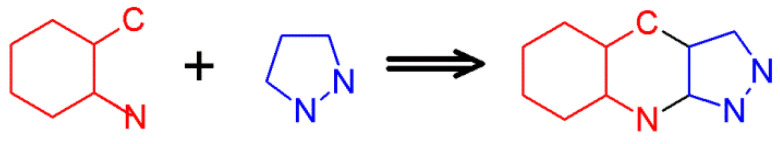
Path 1: C2_1_: C4-C3a; N9-C9a.

**Scheme 1:**
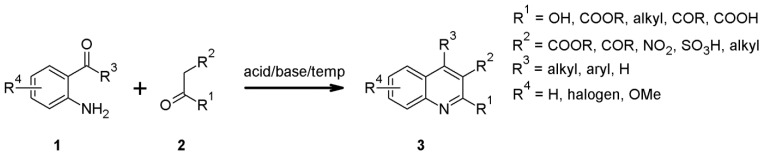
The Friedländer condensation leading to quinoline derivatives.

The Friedländer condensation of *o*-aminocarbonyl compounds **1** (R^3^ = H, alkyl, aryl) with carbonyl systems that contain the active α-methylene group **2** is one of the most important methods of quinoline **3** synthesis [8,9,10,11,12,13] (Figure 4) (Scheme 1).

**Scheme 2:**
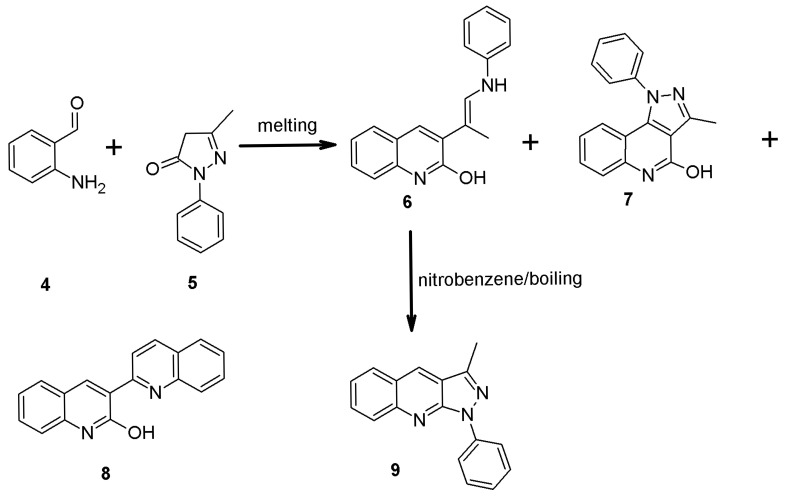
The first attempt at the Friedländer synthesis of 1*H*-pyrazolo[3,4-*b*]quinolines [2].

The reaction can be catalysed by acids and bases, but it also takes place in an inert environment. We start the description of synthetic PQ methods with the oldest results that were published by Niementowski and colleagues, who used the Friedländer condensation of anthranilaldehyde **4** and 5-methyl-2-phenyl-4*H*-pyrazol-3-one **5** (Scheme 2) [2]. As a result, three products were obtained: phenylhydrazone of 3-acetylquinolone **6**; 3-methyl-1-phenyl-1*H*-pyrazolo[4,3-*c*]quinoline **7**; and 3-(2-quinolyl)-quinolin-2-ol **8.** The heating of phenylhydrazone **6** in the boiling of C_6_H_5_NO_2_ led to the formation of 1-methyl-3-phenyl-1*H*-pyrazolo[3,4-*b*]quinoline **9**. Thus, this compound can be described as the first synthesised pyrazoloquinoline.

**Scheme 3:**
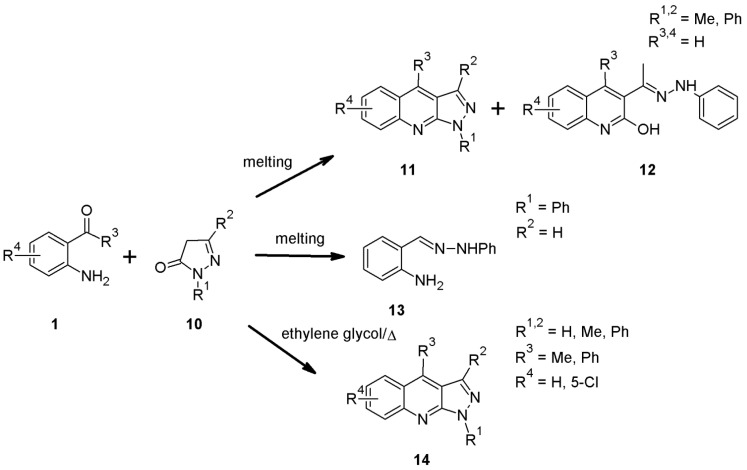
Friedländer-based synthesis of 1*H*-pyrazolo[3,4-*b*]quinolines—an extension.

As for further research on the use of the Friedländer condensation for the synthesis of PQs, a mention should be made of the work of Tomasik et al., which describes a complete synthesis that uses nine pyrazolones **10** substituted with the methyl, phenyl and hydrogen groups with *o*-aminobenzaldehyde **4** (Scheme 3) [14].

The syntheses were conducted within 150–260 °C in melt. The reaction mixtures were separated by column chromatography or crystallisation. PQs **11** were obtained in the cases of five pyrazolones (**10**: R^1,2^ = Ph; R^1,2^ = Me; R^1^ = Me/R^2^ = Ph; R^1^ = H/R^2^ = Me, Ph). In the case of 1,3-dimethylpyrazol-5-one (**10**: R^1,2^ = Me), it was possible to isolate the intermediate product, which was the 4-benzylidene derivative, which suggests that the first step in some of these reactions is the reaction between the aldehyde group and the α-methylene group of the pyrazolone. In the next step, the pyrazolone ring is opened and compound **12** is formed, which then cyclizes to form pyrazoloquinoline **11**. In other cases, 3-acylquinoline alkyl/arylhydrazones **12** or other products were obtained, such as **13** (ca. 100%) in the case when pyrazolone **10** (R^1^ = Ph, R^2^ = H) is used.

Paczkowski et al. investigated pyrazolo[3,4-*b*]quinolines as potential photoinitiators for free radical polymerisation. They prepared these compounds via Friedländer condensation that was conducted in boiling glacial acetic acid [15].

Danel, as an *o*-aminocarbonyl component **1**, applied *o*-aminoacetophenone, *o*-aminobenzophenone and 5-chloro-2-aminobenzophenone, and a complete set of pyrazolones substituted with methyl, phenyl and hydrogen **10** (R^1,2^ = H, Me, Ph) (Scheme 3) [16]. The reaction was carried out in boiling ethylene glycol. Contrary to the two previously mentioned syntheses, in this case, all pyrazoloquinolines **14** were obtained in a single stage, although with different yields (20–85%). Sabitha et al. applied a microwave-assisted condenstion of **1** (R^3^ = H, Me; R^4^ = H) and **10** (R^1^ = Ph, R^2^ = Me) that was supported on clay, which led to 1*H*-pyrazolo[3,4-*b*]quinolines [17].

As mentioned in the introduction, a significant part of pyrazoloquinolines exhibit intense fluorescence, which makes them good luminophores for the fabrication of electroluminescent devices. However, high luminescence efficiency is only one of the many conditions that must be met by candidates for luminophores. The others are a high thermal stability and a high glass-transition temperature. This can be achieved by using spiro compounds, which are just such systems [18]. The introduction of the 9,9’-spirobifluorene system increases the stiffness of the molecule and prevents packing and the intermolecular interactions in the film (after vacuum sputtering on the ITO anode), which hinders crystallisation and increases the Tg value. The tetrahedral nature of the carbon at the centre of the spiro of the system that links the two coupled systems serves as a breaking fragment for this coupling so that the optical and electronic properties of the system are preserved [19].

**Scheme 4:**
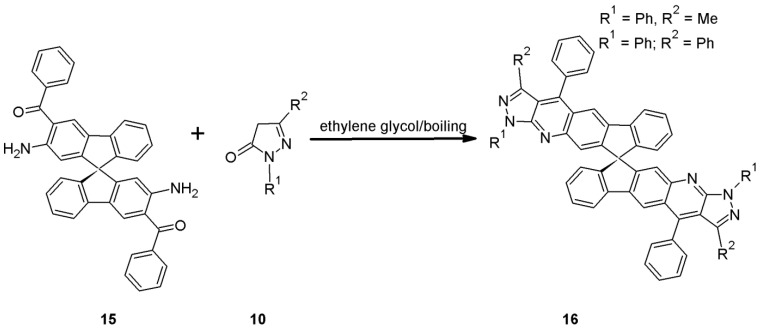
Synthesis of spirobifluorene-based 1*H*-pyrazolo[3,4-*b*]quinolines.

Tao et al. synthesised two spiro PQs **16** systems by using the Friedländer condensation of *o*-aminobenzophenones on the basis of spirobifluorene **15** (Scheme 4) [20].

### 2.2. The Friedländer Synthesis Based on Pyrazole Derivatives

**Figure 5:**
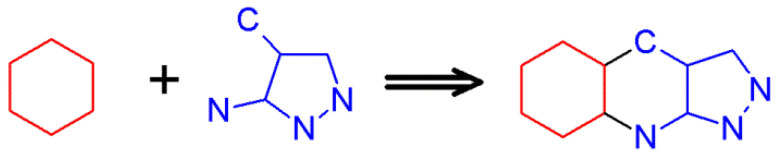
Path 2: C2_2_: C4-C4a; N9-C8a.

**Scheme 5:**
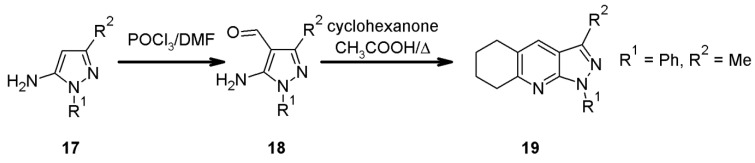
1*H*-Pyrazolo[3,4-*b*]quinoline synthesis from 2-amino-4-pyrazolecarbaldehyde.

**Scheme 6:**
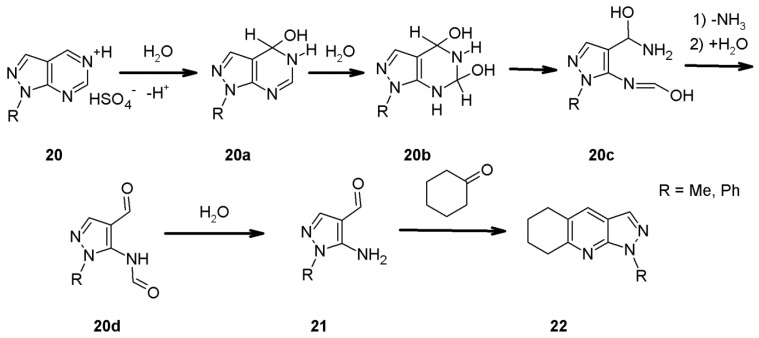
1*H*-Pyrazolo[3,4-*b*]quinoline synthesis from pyrazolo[3,4-*d*]pyrimidine derivatives.

The previously mentioned Friedländer condensation procedures included either the appropriate aldehyde or ketone attached to a carbocyclic ring. One drawback of this synthesis is that the work of obtaining *o*-aminocarbonyl systems **1** is quite troublesome, and aldehydes are often not very stable reagents [21]. The reverse approach is also possible, where pyrazole *o*-aminoaldehyde **18** is used (Figure 5) (Scheme 5 and Scheme 6).

Breitmaier and Häuvel prepared pyrazole aldehyde **18** by the formylation of 5-amino-3-metyl-1-phenylpyrazole **17**. This aldehyde **18** was condensed with cyclohexanone in glacial acetic acid, which yielded 5,6,7,8-tetrahydro-1*H*-pyrazolo[3,4-*b*]quinoline **19** (Scheme 5) [22].

Instead of cyclohexanone, other cyclic ketones can be applied [23]. Higashino et al. prepared pyrazoloquinoline **22** by using aldehyde **21** that was prepared from 1*H*-pyrazolo [3,4-*d*]pyrimidine salts **20** and reactive carbonyl compounds, such as cyclohexanone/cyclopentanone with ethoxide ion as a catalyst (Scheme 6) [6].

The authors propose a number of structures (**20a**–**d**) that can form as intermediates, but they have not isolated any of them. Aldehyde **21** can also be prepared by reducing 5-amino-1-methyl(phenyl)-1*H*-pyrazole-4-carbonitrile with Raney-nickel alloy and formic acid [24].

### 2.3. 1H-Pyrazolo[3,4-b]quinoline Syntheses Based on Anthranilic Acid and Anthranilic Acid Derivatives

Path 1: C2_1_: C4-C3a; N9-C9a (Figure 4)

**Scheme 7:**
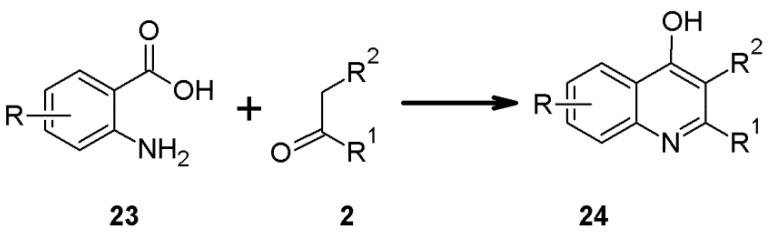
Niementowski’s quinoline synthesis.

Another classic method of synthesising the quinoline **3** is the Niementowski reaction, in which anthranilic acid **23** and ketones **2** (or aldehydes) are used. As a result, 4-hydroxyquinolines **24** are formed (Scheme 7) [25].

**Scheme 8:**
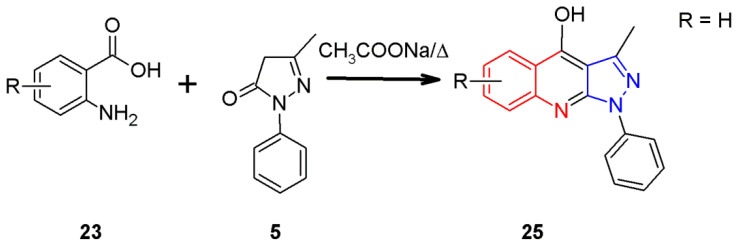
An attempt to apply anthranilic acid for 4-hydroxy-1*H*-pyrazolo[3,4-*b*]quinoline synthesis.

The first attempt to synthesise pyrazoloquinoline by using Niementowski’s synthesis was made by Ghosh in 1937 through the reaction of anthranilic acid **23** and pyrazolone **5** in the presence of anhydrous CH_3_COONa. The product of the reaction was to be 4-hydroxy-3-methyl-1-phenyl-1*H*-pyrazolo[3,4-*b*]quinoline **25** (Scheme 8) [26].

**Scheme 9:**
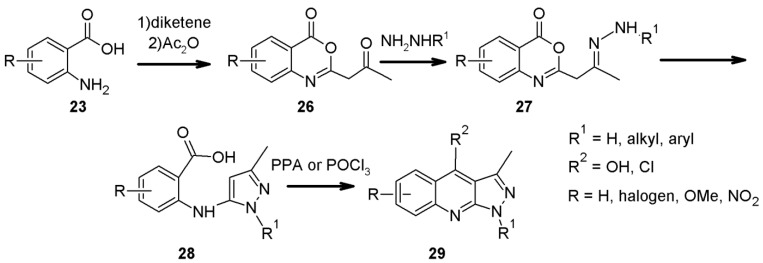
Indirect use of anthranilic acid in synthesis of 1*H*-pyrazolo[3,4-*b*]quinolines.

The reaction was re-examined by Tomasik and co-workers, and it turned out that compound **25** does not arise under these conditions. In the reaction mixture, only traces of two 1*H*-pyrazolo[3,4-*b*]quinolines were found: 1-phenyl-3-methyl-1*H*-pyrazolo[3,4-*b*]quinoline (**14**; R^1^ = Ph, R^2^ = Me, R^3,4^ = H) and 1-phenyl-3,4-methyl-1*H*-pyrazolo[3,4-*b*]quinoline (**14**; R^1^ = Ph, R^2,3^ = Me, R^4^ = H); however, both of them were formed as byproducts. 4-Hydroxy derivative **25** was not formed in this reaction. To prove it, the compound was obtained by another synthetic method, and it was compared to the obtained products by using TLC (Scheme 9); it was not detected in the reaction mixture, according to the Gosh procedure [27,28].

After studying a large number of publications on the synthesis of the compounds that are the subject of this review, it seems that, so far, it has not been possible to obtain pyrazoloquinolines by the direct reaction of anthranilic acid and pyrazolones.

On the other hand, anthranilic acid **23** and its derivatives, in turn, are a valuable substrate for the indirect synthesis of pyrazoloquinolines **29** (Scheme 9). Anthranilic acid **23** is reacted with diketene and acetic anhydride, which yields 2-acetonyl-4*H*-3,1-benzoxazin-4-one **26**, which is easily separated from a reaction mixture by filtration. In the next step, **26** is converted to the pyrazole derivative **28** by reaction with the appropriate hydrazine. There is no need to isolate the intermediate **27**. The final step in this reaction sequence is heating **28** in polyphosphoric acid (PPA) or phosphorus oxychloride (POCl_3_), depending on whether we want to obtain the 4-hydroxy derivative or the compound that contains the chlorine atom in the 4 position (**29**: R^2^ = OH or R^2^ = Cl).

**Scheme 10:**
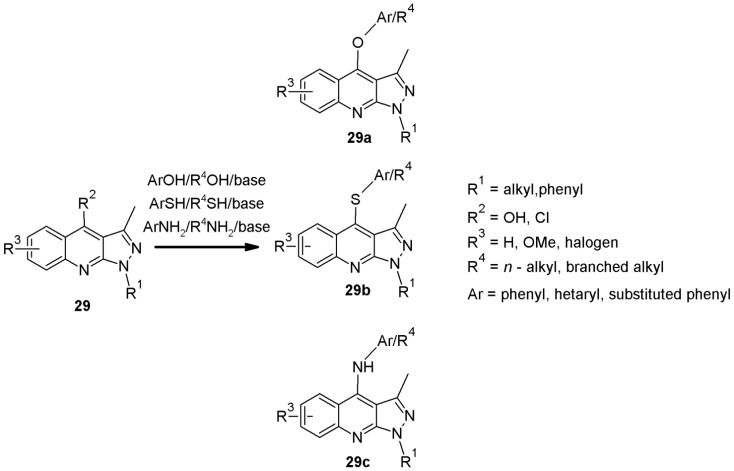
4-chloro/hydroxy derivatives of pyrazoloquinolines as a source for biologically active compounds.

This reaction cycle was used by Stein et al. and by Crenshaw et al. to prepare 4-hydroxy and 4-chloro derivatives (**29**: R^2^ = OH or Cl), which were then used to synthesize a whole range of compounds with biological activity, which will be discussed later in this review [29,30]. The 4-chloro derivatives **29** (R^2^ = Cl) were then reacted with phenols, thiophenols and amines, which yielded phenoxy **29a**, thiophenoxy **29b** and amine-substituted **29c** derivatives. These compounds showed antimalarial, antibacterial or interferon-inducing effects (Scheme 10).

**Scheme 11:**
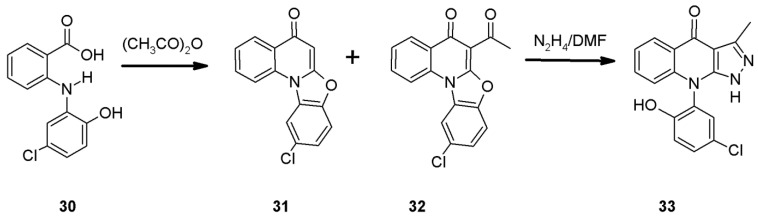
*N*-Arylamide of anthranilic acid as a source for pyrazolo[3,4-*b*]quinoline synthesis.

In the previous reaction, acetic anhydride was the reagent in one of several steps where anthranilic acid played an important role. On the other hand, Kim used it to cyclise *N*-(2-hydroxyphenyl)anthranilic acid **30**, which resulted in two derivatives: **31** and a very small amount of **32** (1.7% yield). The reaction of **32** with hydrazine hydrate yielded a derivative of 1,9-dihydro-3-methyl-4*H*-pyrazolo[3,4-*b*]quinoline-4-one **33** (Scheme 11) [31].

**Scheme 12:**
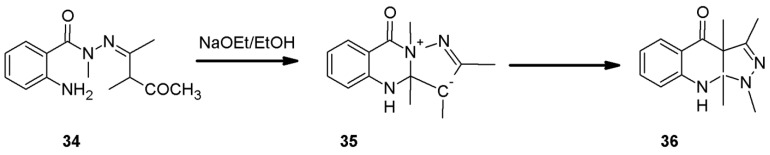
Hydrazide of anthranilic acid as a precursor for pyrazolo[3,4-*b*]quinoline synthesis.

In concluding the discussion of the use of anthranilic acid derivatives, one could also mention the publication of Gal et al., which concerns the transformation of the modified hydrazides of acid **34**. Different products were formed, depending on the reaction conditions (e.g., **35**). When the reaction was run in the presence of sodium ethoxide, the product was pyrazolo[3,4-*b*]quinoline **36** (Scheme 12) [32].

Contrary to the reaction that is shown in Scheme 10, the last two reactions (Scheme 11 and Scheme 12) have no preparative significance.

### 2.4. 1H-Pyrazolo[3,4-b]quinoline Syntheses Based on 4-amino-3-carboxypyrazole Derivatives

Path 2: C_2,2_: C4-C4a; N9-C8a (Figure 5)

**Scheme 13:**
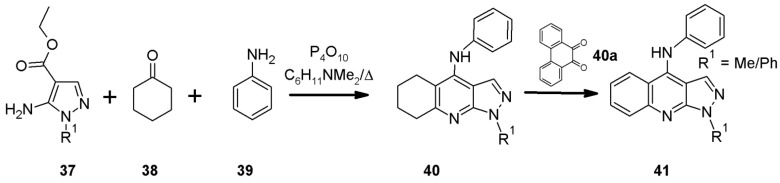
Synthesis of pyrazolo[3,4-*b*]quinoline based on ethyl ester of 3-amino-4-carboxyethylpyrazole.

As in the case of the Friedländer condensation, in this case as well, the derivatives of pyrazoles, such as 5-amino-1*H*-pyrazole-4-carboxylic acid derivatives **37**, can be used. Pedersen obtained 4-*N*-phenyl-1*H*-pyrazolo[3,4-*b*]quinoline **40** by reacting pyrazole derivative **37**, cyclohexanone **38** and aniline **39** in the presence of phosphorus pentoxide and *N*,*N*-dimethyl-*N*-cyclohexylamine. This reaction looks similar to a multicomponent one, but all of the atoms that make up the backbone matrix come only from **37** and **38**. The next step was to oxidise 5,6,7,8-tetrahydro-1*H*-pyrazolo[3,4-*b*]quinolin-4-amine **40** to a fully aromatic **41** with 9,10-phenantrenoquinone **40a** (Scheme 13) [33,34].

**Scheme 14:**
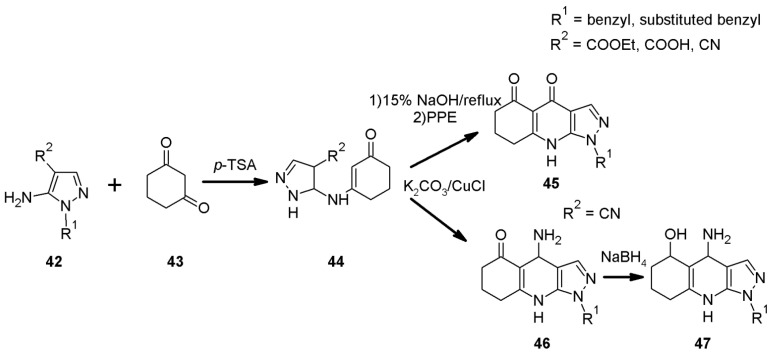
3-Amino-4-pyrazolo carbocylic acid and its ester as a source for pyrazoloquinoline synthesis.

One of the research groups, which was looking for new drugs for the treatment of Alzheimer’s disease, prepared a series of 5,6,7,8-tetrahydropyrazolo[3,4-*b*]quinoline derivatives, such as **45** and **46**, which were then transformed into other systems (e.g., **47**) [35]. Aminopyrazoles **42** were prepared from the appropriate benzylhydrazines and ethoxymethylenemalononitrile (**42**: R^2^ = CN) or ethyl ethoxymethylenecyanoacetate (**42**: R^2^ = COOEt). In the next turn, they were reacted with 1,3-cyclohexanedione **43** in the presence of *p*-toluenesulfonic acid (*p*-TSA). As a result, enamino ketones **44** were formed. In the case of the enemino ketone **44**, where R^2^ was a COOEt substituent, the compound was hydrolysed to produce a derivative with the COOH carboxyl group (**44**: R^2^ = COOH). Next, cyclisation was performed in the presence of polyphosphoric esters (PPE) afforded **45**. The use of potassium carbonate and copper(I) chloride in the case of **44** (R^2^ = CN) promotes the formation of **46**, respectively (Scheme 14).

**Scheme 15:**
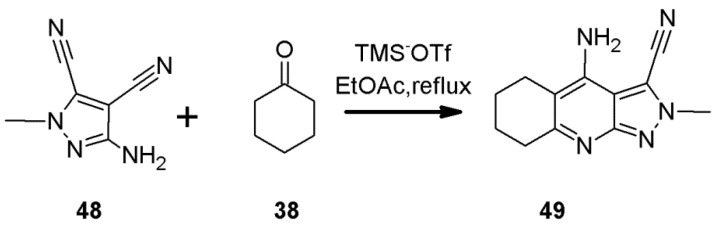
5-Amino-2-methyl-pyrazole-3,4-dicarbonitrile in the synthesis of pyrazolo[3,4-*b*]quinoline derivatives.

Campbell and Firor prepared pyrazolo[3,4-*b*]quinolines **46** by cadmium-chloride- or copper-acetate-promoted cyclisations of pyrazolo enaminones **44** (R^2^ = CN, R^1^ = alkyl) [36]. Instead of toxic cadmium chloride, zinc chloride can also be used [37]. The system with the carbocyclic ring without any functional group can be prepared by starting from cyclohexanone **38** and 4-amino-1-methyl-pyrazole-2,3-dicarbonitrile **48** (Scheme 15) [38].

The resulting 2*H*-pyrazolo[3,4-*b*]quinoline-3-carbonitriles **49** was subjected to further transformations in order to find new pharmacologically active compounds.

### 2.5. The Pfitzinger Synthesis of 1H-pyrazolo[3,4-b]quinolines

Path 1: C2_1_: C4-C3a; N9-C9a (Figure 4)

**Scheme 16:**
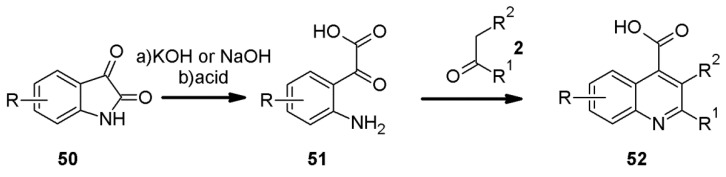
The Pfitzinger synthesis of quinoline.

One of the classic named chemical reactions that leads to the quinoline system is the Pfitzinger synthesis [39,40]. It uses isatin or its derivatives **50**, which are easier to obtain than *o*-aminobenzaldehydes. The reaction is carried out in a basic environment, at which point the opening of the heterocyclic ring takes place to form a keto-acid **51**, which is then condensed with aldehydes/ketones **2**. Additionally, the final product, **52**, can be decarboxylated (Scheme 16).

**Scheme 17:**
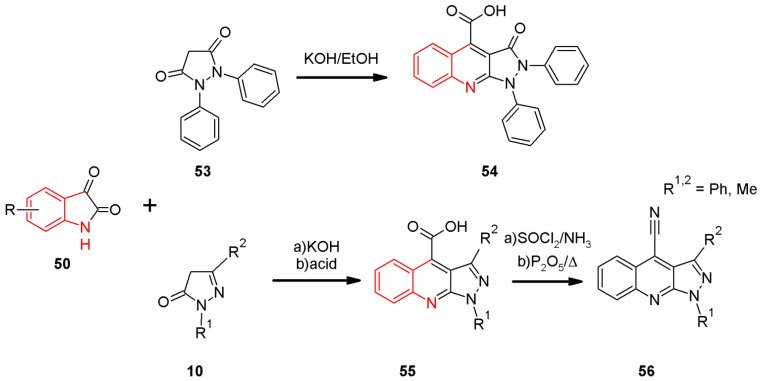
Synthesis of pyrazolo[3,4-*b*]quinolines from isatine.

The use of isatin **50** for the synthesis of pyrazolo[3,4-*b*]quinolines, where this system is built into the backbone, is very limited. One of the reactions is the synthesis that is reported by Fabrini [41]. A reaction between isatine **50** and 1,2-diphenylpyrazolidine-3,5-dione **53** afforded 1*H*-pyrazolo[3,4-*b*]quinolin-3-one-4-carboxylic acid **54**. In a similar fashion, Seshadri combined isatin **50** and 1,3-disubstituted pyrazolin-5-ones **10** under basic conditions and obtained 4-carboxylic acids **55**, and with nitriles 56 after subsequent reactions of **55** with SOCl_2_ and P_2_O_5_ (Scheme 17) [42].

Recently, a few studies have appeared on multicomponent pyrazoloquinolines syntheses where isatins and their derivatives are used. The end product is the *spiro*-type systems, where isatin brings C-4 carbon to the overall structure. These reactions will be discussed in the final section on the synthesis of this class of compounds.

### 2.6. 1H-Pyrazolo[3,4-b]quinoline Syntheses Based on 5-chloro-4-aroyl/formylpyrazoles and 4-benzylidene-1,3-disubstituted-pyrazol-5-ones

**Figure 6:**
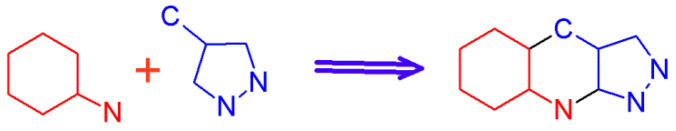
Path 3: C2_3_: N9-C9a; C4-C4a.

Another method of pyrazoloquinolines synthesis is the use of aromatic amines and 4 substituted pyrazole derivatives such as aldehydes, ketones or benzylidene ones (Figure 6).

**Scheme 18:**
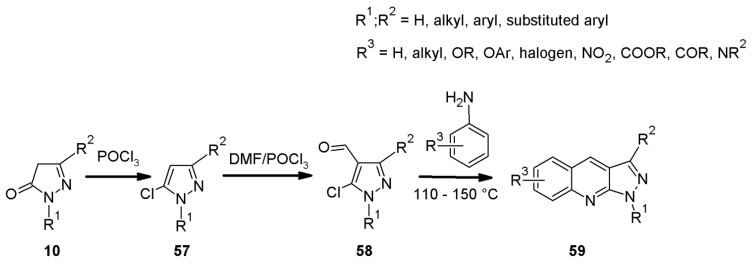
Synthesis of 1*H*-pyrazolo[3,4-*b*]quinolines, according to Brack’s protocol.

In 1963, Brack published a work on the synthesis of pyrazoloquinolines that was based on the reactions of aromatic amines **39** and 5-chloro-4-formylpyrazoles **58** [43,44]. The latter compounds were obtained by the formylation of the appropriate 5-chloropyrazoles **57** [45]. They can also be obtained in one step in the Vilsmeier–Haack reaction by treating the pyrazolones **10** with a DMF / POCl_3_ mixture [46]. The original Brack’s reaction was carried out in melt within 110–150 °C (Scheme 18).

**Scheme 19:**
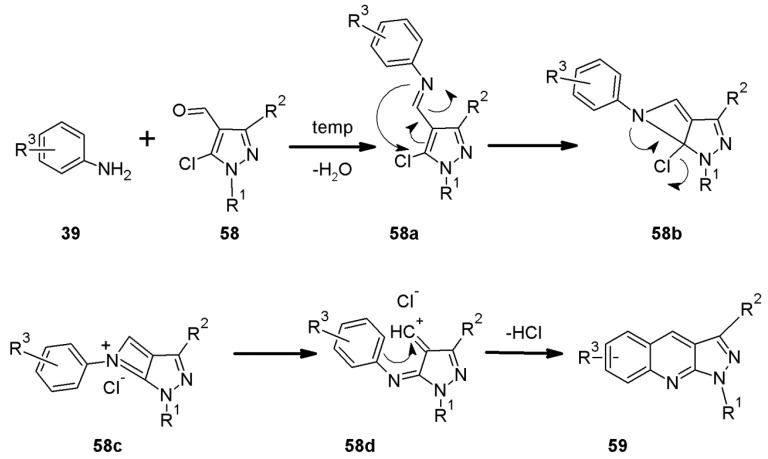
The possible mechanism of Brack’s synthesis.

Two possible schemes for this reaction have been found in the literature. One was proposed by Brack (Scheme 19). The reaction starts from the formation of Schiff base **58a** from aniline **39** and aldehyde **58**. In the next steps, several rearrangements take place with the formation of the final pyrazoloquinoline **59**.

**Scheme 20:**
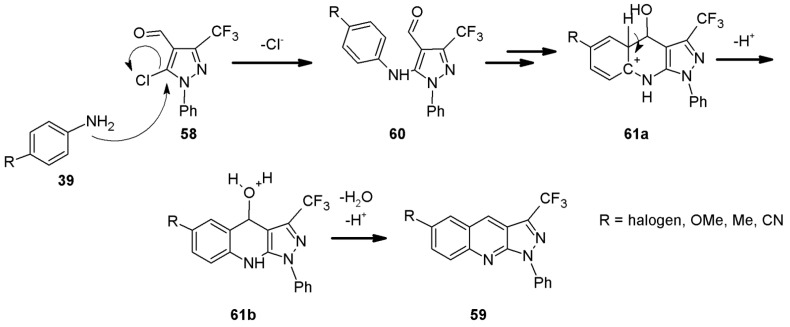
An alternative mechanism of Brack’s 1*H*-pyrazolo[3,4-*b*]quinoline synthesis.

Another mechanism was proposed a few years ago that consists of the nucleophilic substitution of the chlorine atom in pyrazole **58** by an amino group in **39** with subsequent ring formation, and the elimination of the water molecule and hydrogen cation, which leads to the aromatisation of the system (intermediates **60-61b**) (Scheme 20) [47]. It seems, however, that the experimental data support the former; therefore, it was possible to isolate the intermediate product, which was Schiff’s base **58a**, which was formed from pyrazole aldehyde **58** and substituted aniline **39**. The authors of the later paper were not able to isolate amine derivative **60**.

**Scheme 21:**
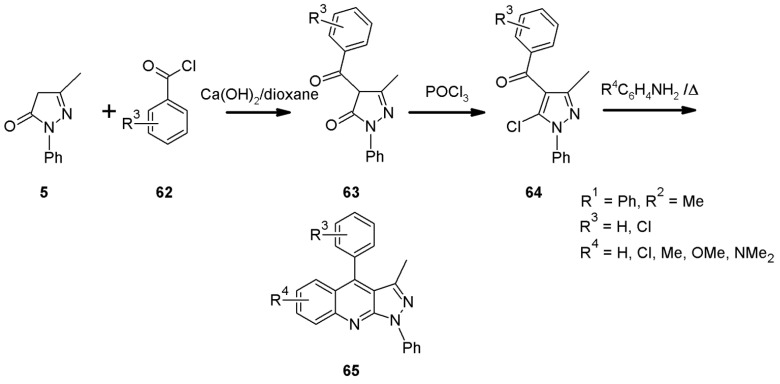
Synthesis of 1*H*-pyrazolo[3,4-*b*]quinolines based on 4-aroyl-5-chloropyrazoles.

In the original work of Brack, the author limited himself mainly to three 5-chloro-4-formylpyrazole aldehydes (**58**; R^1^ = Me, R^2^ = Ph; R^1^ = Ph, R^2^ = Me; R^1^ = tetrahydro-1,1-dioxo-3-thienyl, R^2^ = Me) and a few aromatic amines. However, this reaction has many more possibilities, as is shown in several later works by other authors [48]. The use of 5-chloro-4-aroylpyrazoles **64** is an extension of the scope of two-component reactions with aromatic amines R^4^C_6_H_4_NH_2_ and pyrazole systems with aldehyde function **58** [49] (Scheme 21).

Pyrazole derivatives **64** can be synthesised either by the acylation of 5-chloropyrazoles with aromatic acid chlorides **62** and aluminum chloride, or by reacting pyrazolones **10** and acid chlorides **62**, followed by chlorination with phosphorus oxychloride [50] (Scheme 21). In contrast to the reaction with aldehydes **58**, this reaction required much more severe conditions and it lasted from 2 to 3 h at 220 °C. In one case, the authors isolated an intermediate product, which was the product of the nucleophilic amine substitution of the aromatic chlorine atom at the 5 position of the pyrazole ring. This proves a different mechanism than in the case of the original Brack reaction with pyrazole aldehyde **58** (Scheme 19), and it would, in a way, confirm the mechanism that was proposed for this reaction by Wan et al. (Scheme 20) [50].

**Scheme 22:**
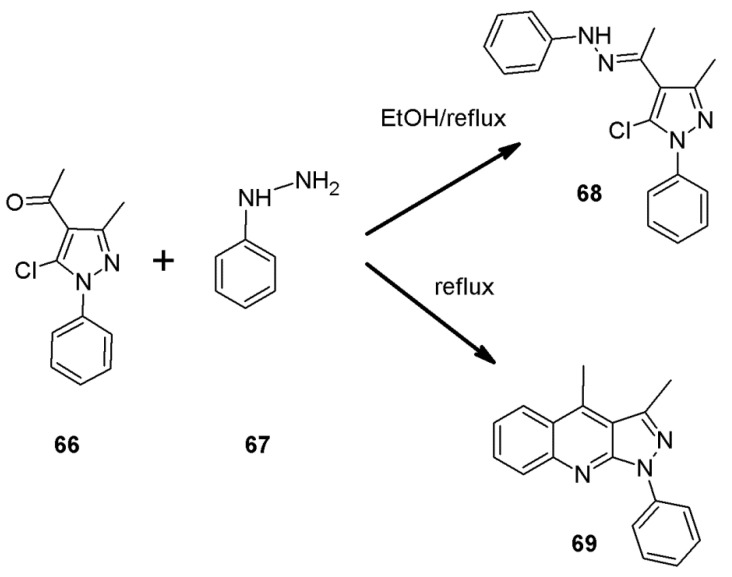
Reaction of phenylhydrazine with 4-acetyl-5-chloro-3-methyl-1-phenylpyrazole.

Gonzales and El Guero probably describe the only reaction where the 4 acetyl derivatives of chloropyrazole **66** and phenylhydrazine **67** were used for the synthesis of pyrazoloquinoline **69**. Depending on the reaction conditions, either the corresponding phenylhydrazone **68** or pyrazoloquinoline **69** are obtained. In the latter case, the authors suggest that phenylhydrazine decomposes under the influence of high temperatures, and the resulting aniline reacts with the chlorine atom with subsequent cyclisation to pyrazoloquinoline [51] (Scheme 22).

**Scheme 23:**
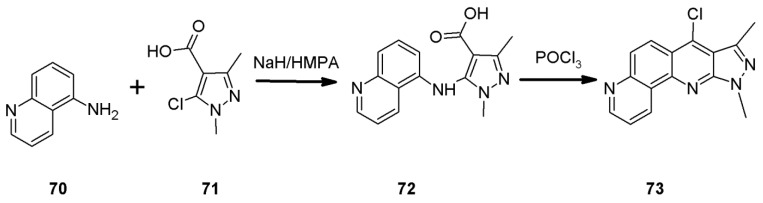
Synthesis of 10*H*-pyrido[2,3-*h*]pyrazolo[3,4-*b*]quinoline.

Crenshaw et al. synthesised 10*H*-pyrido[2,3-*h*]pyrazolo[3,4-*b*]quinoline **73** by starting from 5-aminoquinoline **70** and 5-chloro-1,3-dimethylpyrazolo-4-carboxylic acid **71** [52] (Scheme 23). The purpose of this reaction was to confirm the structure of product **73** that was unexpectedly formed by the ring closure of the derivative **28** (R^1^ = Me, R = 4-NO_2_), instead of the expected 4-chloro-7-nitro-1,3-dimethyl-1*H*-pyrazolo[3,4-*b*]quinoline **29** (R^1^ = Me, R^2^ = Cl, R = 7-NO_2_) (Scheme 9).

Regardless of the analysis of the structure of compound **73** by using the ^1^H NMR spectrum, the authors synthesised it by reactions of aminoquinoline **70** and pyrazole acid **71** in the presence of sodium hydride, and they then cyclised the intermediate **72** with POCl_3_.

**Scheme 24:**
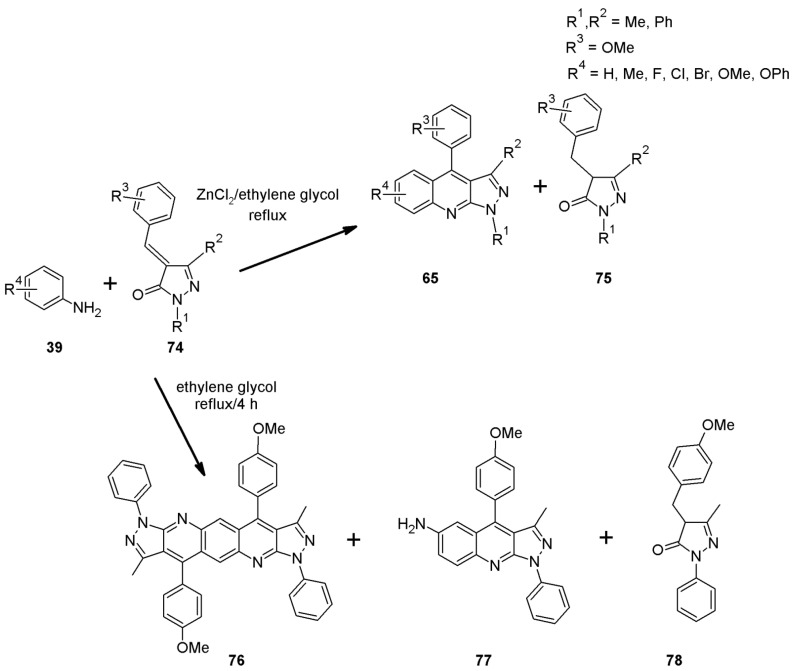
Synthesis of pyrazolo[3,4-*b*]quinolines from 4-benzylidenepyrazol-3-ones and anilines.

Tomasik et al. report a new method toward 1*H*-pyrazolo[3,4-*b*]quinolines that uses anilines **39** and benzylidene derivatives **74** (Scheme 24). This reaction produces derivatives that are substituted with a phenyl group (or a substituted phenyl group) in position 4 of the parent backbone **65**. As a side product, compound **75** was isolated. It is an excellent alternative to Friedländer condensation, where 2-aminobenzophenones **1** (R^3^ = Ph) are used (Scheme 3) [1,53].

While searching for the literature on the synthesis of pyrazoloquinolines, we came across a work where a whole series of syntheses of various pyrazole derivatives with potential biological effects were described [54]. Among them was the synthesis that uses the benzylidene derivative **74** (R^1^ = Ph, R^2^ = Me, R^3^ = *p*-OMe) and 1,4-phenylenediamine **39** (R^4^ = *p*-NH_2_) (Scheme 24). The authors took into account the possibility of the formation of a condensed **76** system, but they did not find it in the reaction products. They only describe that they received compound **77**, which was supposed to be a pyrazoloquinoline and a side product **78**. However, they did not provide evidence of this in the form of ^1^H NMR and ^13^C NMR spectra for both of them. The obtained substance (**77**) was white, which is in contradiction to our research to date. We have obtained a whole range of variously substituted 1,4-diphenyl-3-methyl-1*H*-pyrazoloquinolines, which are always yellow crystalline substances, and are, in addition, also strongly fluorescing, which the authors of the publication did not mention. Thus, it seems to us that they failed to obtain **77**.

### 2.7. 1H-Pyrazolo[3,4-b]quinoline Syntheses Based on Aminopyrazoles

**Figure 7:**
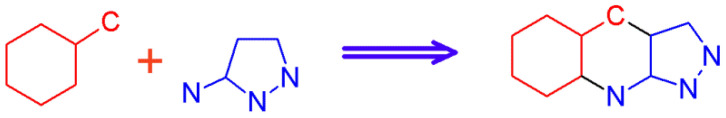
Path 4; C2_4_: C4-C3a; N9-C8a.

The procedure that was developed by Brack is an extremely effective tool for the synthesis of pyrazoloquinolines. There is also the possibility of using substituted *o*-halogenobenzoic aldehydes and aminopyrazoles (Figure 7).

**Scheme 25:**
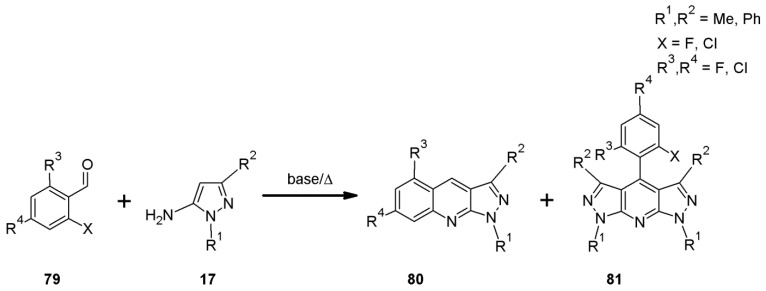
Synthesis of pyrazolo[3,4-*b*]quinolines from 3-aminopyrazoles and halogenated aromatic aldehydes.

Szlachcic et al. have published a very extensive work on the use of variously substituted *o*-halogeno benzaldehydes **79** for the synthesis of pyrazoloquinolines **80** [55] (Scheme 25). The authors focused on regioselective reactions, which would allow the preparation of mono- and disubstituted pyrazoloquinoline halogen derivatives, which could be used later for further reactions (e.g., nucleophilic substitution with aliphatic or aromatic amines), and which could be used, for example, as luminophores for the construction of electroluminescent cells.

In the case of monosubstituted aldehydes **79** (R^3,4^ = H, X = F, Cl, Br, I), it has been observed that, besides pyrazoloquinoline **80**, *bis*-pyrazolo[3,4-*b*;3′,4′-e]pyridine **81** is also formed; however, in the case of *o*-fluorobenzaldehyde, pyrazoloquinoline was the final product. The opposite tendency was observed for *o*-iodobenzaldehyde **79** (R^3,4^ = H, X = I). Pentafluorobenzaldehyde yielded only pyrazoloquinoline **80**. As part of the work, the influence of the base on the course of the reaction was also investigated, and it turned out that bases such as DABCO or quinoline favored the formation of **80** but not of **81**.The influence of the substituents in the aminopyrazole **17** was also of some importance, and so the system with the phenyl group in position 3 (**17**; R^2^ = Ph) favored the formation of PQ, while the methyl group contributed to the formation of more **81**. Dehaen et al. investigated the reaction pentafluorobenzaldehyde and 5-chloro-4-formylpyrazoles **17** with 5-amino-1,2-azoles. Depending on the reaction conditions, the positions of the halogen atom in relation to the carbonyl group pyrazolo[3,4-*b*]quinolines **80**, *bis*-pyrazolo[3,4-*b*; 4′,3′-*e*]pyridines **81** and isoxazolo[5,4-*b*]quinolines were formed [56].

**Scheme 26:**
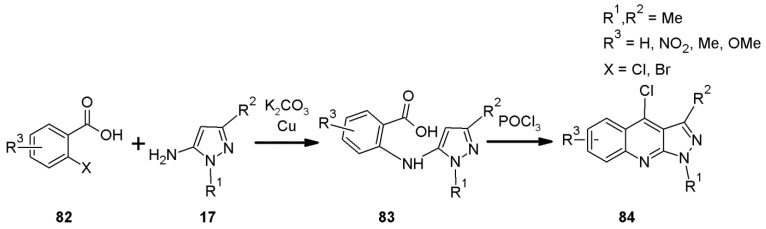
Synthesis of pyrazolo[3,4-*b*]quinolines from 3-aminopyrazoles and orto-chloro/bromo benzoic acid.

Another reaction route is the use of *o*-halogen-substituted benzoic acids **82** and 1,3-disubstituted 5-aminopyrazoles **17.** As a result of the Ullmann-type reaction that is catalysed by copper, an intermediate **83** is formed. In the next step, it can be transformed into **84** by heating with POCl_3_ (Scheme 26). This reaction was used, inter alia, by Siminoff and other researchers for the synthesis of **84** from anthranilic acid **23** and diketene [34,57,58] (Scheme 9).

**Scheme 27:**
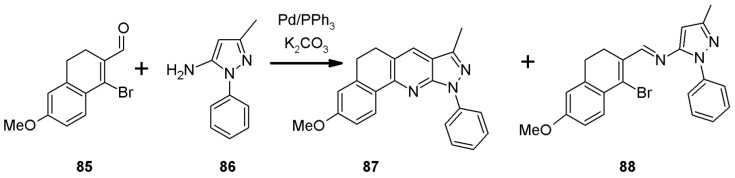
Application of *β*-bromovinyl aldehyde in synthesis of 1*H*-pyrazolo[3,4-*b*]quinolines.

Boruah et al. employed a palladium catalysed reaction of *β*-bromovinyl aldehyde **85** with **86**, which yielded pyrazolo[3,4-*b*]quinoline **87** and Shiff base **88** (Scheme 27) [59].

The ratio of the products that were obtained depended on the conditions under which the reaction was carried out. Thus, during heating at 120 °C for 24 h, the Schiff’s base **88** dominated in the reaction mixture, while the reaction that was carried out in the microwave field, without a solvent, for 15 min, favored the formation of pyrazoloquinoline **87**. As a catalyst, 2.5 mol% palladium acetate has been proven to be the best.

**Scheme 28:**
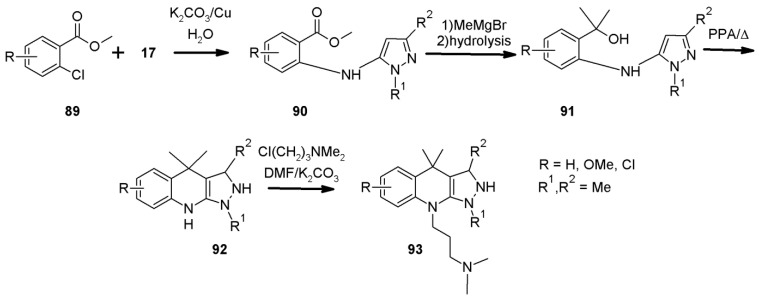
Benzyl alcohol derivatives as a substrate for pyrazolo[3,4-*b*]quinoline synthesis.

At the end of the description of the use of the *o*-halogen derivatives of acids, esters and aldehydes, we want to mention the use of a benzyl alcohol derivative that has been cyclised to pyrazoloquinoline. In 1974, Horace de Wald synthesised a series of pyrazoloquinolines **93** as potential antidepressants [60]. As a starting material, he applied the methyl ester of 2-chlorobenzoic acid **89**. The Ullmann reaction with 1,3-dimethyl-5-amino pyrazole **17** afforded the formation of **90**. This compound was reacted with Grignard reagent CH_3_MgBr, and it was hydrolysed to yield an alcohol **91**, which was cyclised to **92** with PPA at 85–110 °C (Scheme 28).

### 2.8. 1H-Pyrazolo[3,4-b]quinoline Synthes Based on Quinoline Derivatives

**Figure 8:**
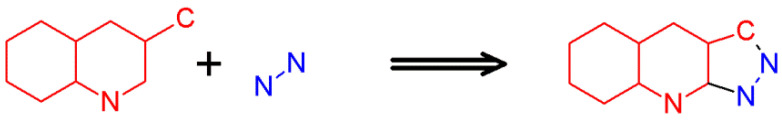
Path 5; C2_5_:N1-C9a; N2-C3.

The most common way to obtain pyrazol*o*[3,4-b]quinolines by using the fifth path is by the reaction of the hydrazines RNHNH_2_ (R = H, aryl) with quinoline derivatives (Figure 8).

**Scheme 29:**
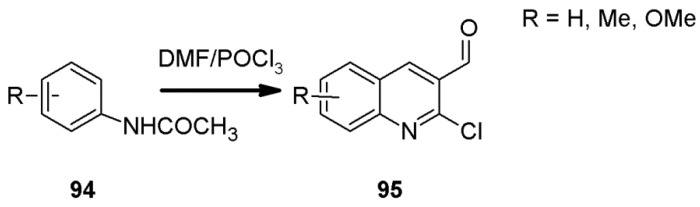
Synthesis of 5-chloro-4-formyloquinolies, according to Meth-Cohn protocol.

Some modifications are also possible, in which 2-chloro/2-aminoquinolino-3-carbonitrile or 3-acetylquinolin-2(1*H*)-one can also be used. In 1978, Meth-Cohn et al. described the synthesis of the 5-chloro-4-formylquinoline derivatives **95** by the formylation of Vilsmeier–Haack-substituted acetanilides **94** (Scheme 29) [61,62,63].

**Figure 9:**
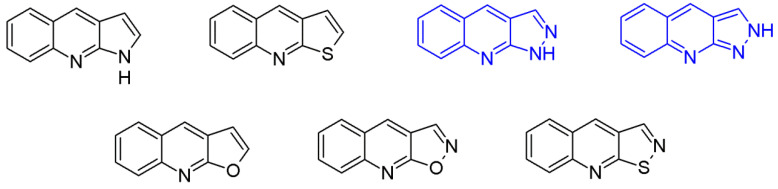
Some heterocycles prepared from 2-chloro-4-formyl quinoline [64].

The resulting compound, 5-chloro-4-formylquinoline **95**, can be transformed into a wide variety of fused heterocyclic derivatives, including 1*H*- or 2*H*-pyrazolo[3,4-*b*]quinolines (Figure 9) [64].

**Scheme 30:**
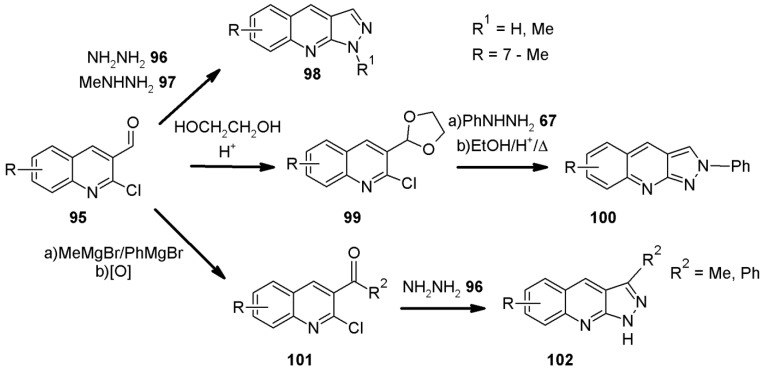
1*H*-Pyrazolo[3,4-*b*]quinoline syntheses from 2-chloro-3-formylquinolines [61].

Some simple procedures of transforming 2-chloro-3-formylquinolines **95** into pyrazoloquinolines are depicted in Scheme 30. Thus, Hayes et al. carried out the reaction between **95** and hydrazine **96** or methylhydrazine **97**. The immediate attack at the aldehyde group forms 1,7-dimethyl-1*H*-pyrazolo[3,4-*b*]quinoline **98**. On the other hand, when the aldehyde group is protected, such as in **99**, hydrazine substitutes the chlorine atom at position 2, and with the subsequent deacetylisation with acid in alcoholic solution and the cyclisation to the pyrazole ring. The formation of 2*H*-pyrazolo[3,4-*b*]quinolines **100** is observed. The third modification consists of reacting the Grignard reagent with an aldehyde group in **95**, and the resulting alcohol is oxidised with chromium (VI) compounds to the ketone **101**. Reaction with hydrazine or arylhydrazine leads to the formation of 1*H*-pyrazolo[3,4-*b*]quinolines **102** [65].

**Scheme 31:**
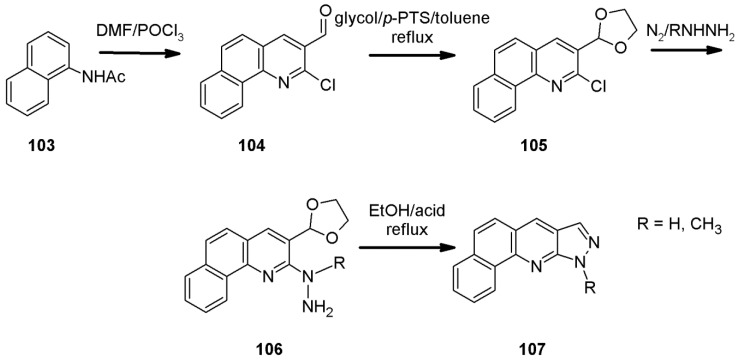
Synthesis of benzo[*h*]pyrazolo[3,4-*b*]quinolines.

Other researchers have applied the same methodologies, and sometimes with the application of MW irradiation [65,66,67,68,69,70,71,72,73,74]. Mane et al. investigated the influence of baker’s yeast on the synthesis of tetrahydrobenzo[*a*]xanthene-11-ones and pyrazolo[3,4-*b*]quinolines. They reacted substituted aldehydes **95** and hydrazine **96** or phenylhydrazine **67** in water and obtained pyrazolo[3,4-*b*]quinolies **98** (R = H, Me, Cl, OMe, R^1^ = H, Ph), with the yield of 79–90% [75]. In 1999, Kerry et al. formylated *N*-acetylo-1-naphthylamine **103** to produce 2-chlorobenzo[*h*]quinoline-3-carbaldehyde **104**. In the next turn, the aldehyde group was protected by transformation into acetal **105** and was reacted with methylhydrazine **97** or hydrazine **96**, which formed compound **106**. This one was heated in boiling ethanol that was acidified with HCl, which afforded 10-methyl-10*H*-benzo[*h*]pyrazolo[3,4-*b*]quinoline **107** (Scheme 31) [76]. These derivatives were synthesised as potential topoisomerase inhibitors.

**Scheme 32:**
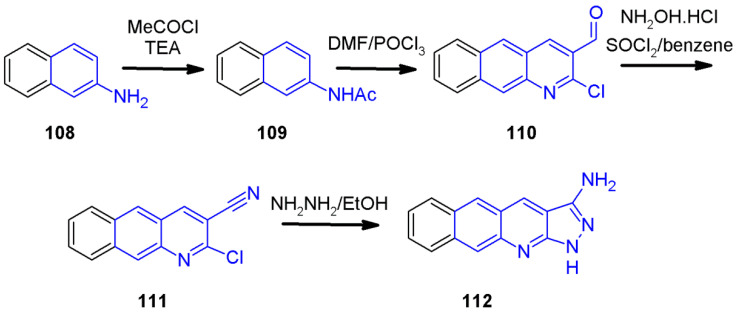
Synthesis of 1*H*-benzo[*g*]pyrazolo[3,4-*b*]quinoline-3-ylamine.

When 2-naphthylamine **108** is used instead of 1-naphthylamine, the aldehyde **110** can be obtained by carrying out the same chemical transformations (e.g., acetyl derivative **109**) as for the previously described reactions. Maheira converted **109** into an oxime by reaction with hydroxylamine hydrochloride and, with the subsequent treatment with SOCl_2_ in boiling benzene, he obtained 2-chloro-benzo[*g*]quinoline-3-carbonitrile **111**. Further reaction with hydrazine **96** gave **112**. In the next turn, 3-aminoderivative **112** was coupled with various pyrazol-5-ones, which formed a series of azo dyes for polyester fibers (Scheme 32) [77,78].

The same synthetic protocol can be used for **95** by the transformation of it into oxime, 2-chloroquinoline-3-carbonitrile and then into 3-amino-1*H*-pyrazolo[3,4-*b*]quinoline, which is then modified in various ways to search for potential compounds of biological activity (Scheme 32—structures marked in blue) [79].

**Scheme 33:**
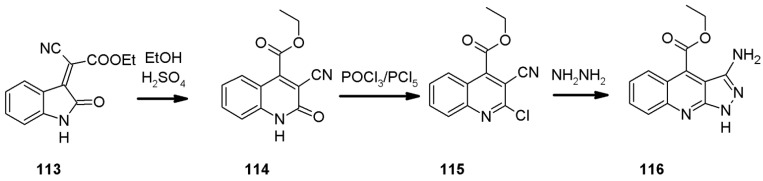
Synthesis of ethyl 3-amino-1*H*-pyrazolo[3,4-*b*]quinoline-4-carboxylate.

Section 2.5 discusses the few cases where isatin and pyrazolones are used in the Pfitzinger reaction for the synthesis of pyrazoloquinolines. Here, we have another one of the few cases that uses this compound, and specifically its derivative **113**, which is formed as a result of the reaction of isatin **50** with ethyl cyanoacetate. The reaction with ethanol acidified with sulphuric acid afforded ethyl 3-cyano-2-oxo-1,2-dihydroquinoline-4-carboxylate **114**. Heating with a mixture of phosphorus oxychloride and phosphorus trichloride causes the introduction of the chlorine atom in the 2 position of the quinoline system, which results in the formation of compound **115**. The last step is the reaction with hydrazine, which gives pyrazoloquinoline **116**. This compound offers multiple possibilities for further functionalization and for obtaining a whole range of derivatives (e.g., by modifying the ester and amino groups, or by adding substituents to the nitrogen atom N1 (Scheme 33) [80]).

**Scheme 34:**
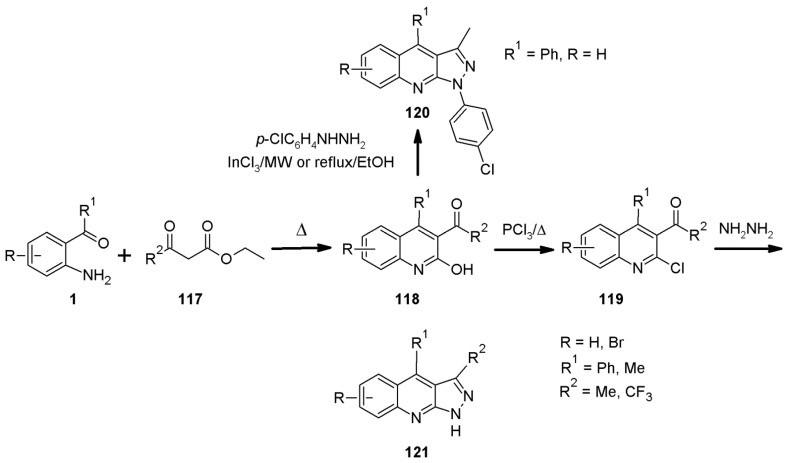
Syntheses of 1*H*-pyrazolo[3,4-*b*]quinolines from 2-hydroxy-3-acylquinolines.

Another substrate for the synthesis of pyrazolo[3,4-*b*]quinolines is 2-hydroxy-3-acetylquinoline **118**, which is easily prepared from ethyl acetoacetate **117** and *o*-aminoacetophenone/benzophenone **1** (Scheme 34). Arasakumar et al. applied 3-acetyl-4-phenyl-chinolin-2-one **118** and *p*-chlorophenylhydrazine for the synthesis of substituted 1*H*-pyrazolo[3,4-*b*]quinoline **120** [81]. Researchers have studied the effects of various Lewis acids (e.g., SnCl_4_, AlCl_3_, TiCl_4_, etc.), as well as of the microwave field, on the reaction yield. The best results were achieved with indium chloride (InCl_3_).

**Scheme 35:**
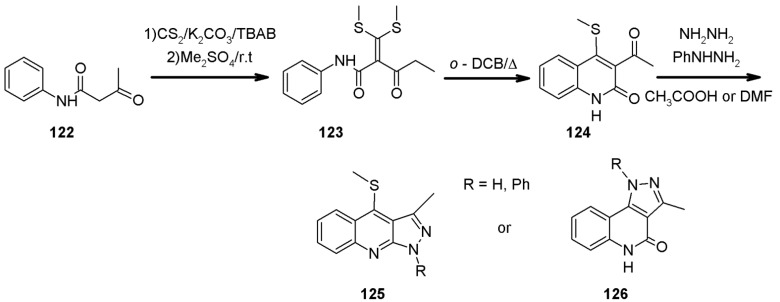
Synthesis of 4-methylthio-1*H*-pyrazolo[3,4-*b*]quinolines.

Compound **118** can be subjected to the reaction with phosphorus trichloride (PCl_3_) to give 2-chloro-3-acetylquinolines **119**, which is reacted with hydrazine. The product is 3,4-disubstituted-1*H*-pyrazolo[3,4-*b*]quinoline **121** [82]. The free position in nitrogen N1 allows for numerous modifications of these compounds in terms of the biological properties [83]. 3-Acetyl-4-(methylthio)quinolin-2(1*H*)-one **124** is an example of another quinolin-2-one-based system that can be used both for the synthesis of pyrazoloquinolines and for other heterocycles as well [84]. It can be easily prepared from acetoacetanilide **122** by reacting with carbon disulfide in the presence of tetrabutylammonium bromide (TBAB), with the subsequent methylation yielding ketene dithioacetal **123**. In the next step, the **123** is cyclised by boiling in *o*-dichlorobenzene. Reactions with hydrazine or phenylhydrazine in acetic acid lead to the formation of the corresponding pyrazoloquinolines **125**. On the other hand, when DMF is used as a reaction medium instead, **125** angular pyrazolo[4,3-*c*]quinolin-2-ones **126** are formed (Scheme 35) [85].

**Scheme 36:**
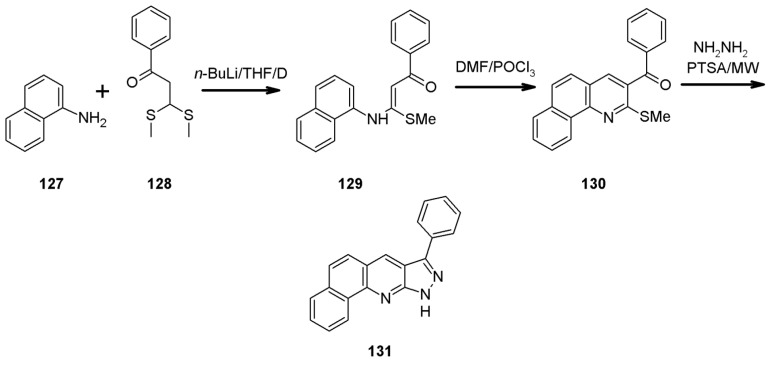
Syntheses of 2-methylthio-3-benzoyl-4-methylquinolines and related 1*H*-pyrazolo[3,4-*b*]quinolines.

The use of 2-naphthylamine **108** and the Meth-Cohn procedure allows for the synthesis of benzo[*h*]pyrazolo[3,4-*b*]quinoline (Scheme 32). Similarly, 1-naphtylamine **127** can be reacted with α-oxoketene dithioacetal **128** and *n*-BuLi to form α-oxoketene -*N*,*S*-naphtyaminoacetal **129**, which forms 2-methylthio-3-benzoyl-4-methylquinolines **130** upon the cyclisation by POCl_3_/DMF. The reaction with hydrazine under microwave irradiation afforded the formation of **131** (Scheme 36) [86].

The same procedure can be applied to aromatic amines or diamines and, as a result, other heterocyclic systems, such as phenanthrolines, can be obtained.

### 2.9. 1H-Pyrazolo[3,4-b]quinoline Syntheses Based on 5-arylaminoaminopyrazoles

**Figure 10:**
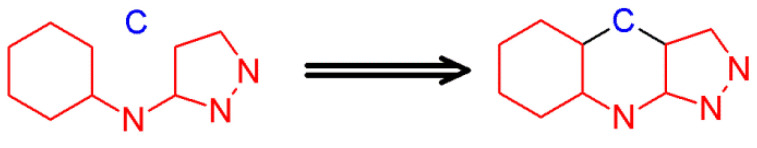
Path 6a; C2_6a_: C4-C4a; C4-C3a.

One of the oldest methods of pyrazoloquinolines synthesis are the two-component reactions of 5-arylamino pyrazoles and aromatic aldehydes (Figure 10).

**Scheme 37:**
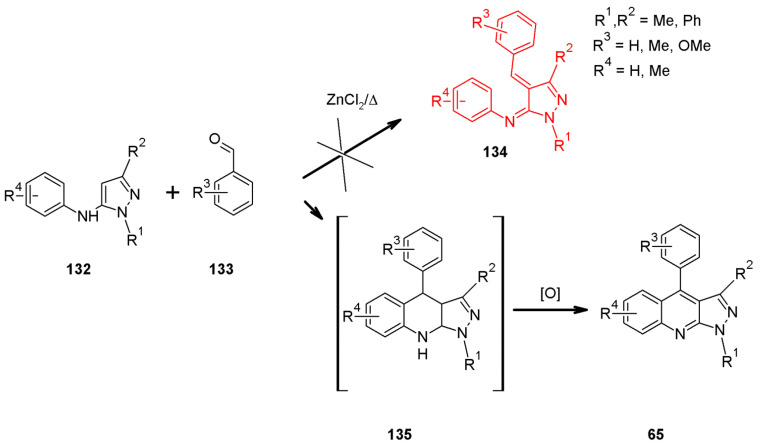
Correction and verification of the Michaelis’ 1*H*-pyrazolo[3,4-*b*]quinoline synthesis.

In 1911, Michaelis heated 5-*N*-arylamino-1,3-disubstituted pyrazoles **132** with some aromatic aldehydes **133** in the presence of anhydrous ZnCl_2_ and obtained compounds to which he assigned a structure **134** (Scheme 37) [1]. He described them as yellow crystalline substances with a strong blue fluorescence in a toluene solution. Our group was interested in these types of compounds from the point of view of applying them to organic electroluminescent cells. The syntheses described by Michaelis were repeated, and the chemical structures of the compounds that were obtained were analysed by using ^1^H NMR spectroscopy. It turned out that the structure of Michaelis **134** did not correspond to any of the compounds that he received, but that it did match the pyrazoloquinolines of **65** [87].

In several cases, it was possible to isolate intermediate **135**, which oxidises over time to form pyrazolo[3,4-*b*]quinoline **65**. The modified Michaelis method turned out to be a very good method for the synthesis of **65**, and it complements the Friedländer condensation, in which *o*-aminobenzophenones are used. However, it provides more possibilities with regard to the introduction of substituents to the phenyl ring in position 4 and the modification of the carbocyclic ring. The starting 5-*N*-arylamino-pyrazoles **132** can be obtained from commercial 1,3-disubstituted 5-aminopyrazoles and the corresponding aryl halides by the procedures that are described by Buchwald [88].

**Scheme 38:**
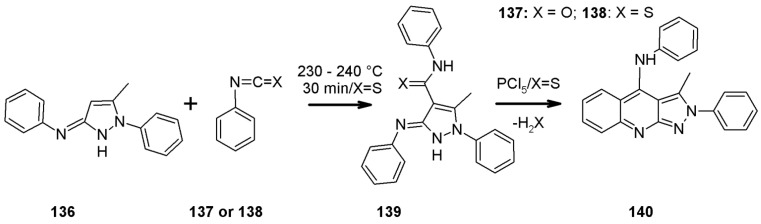
Reactions of isocyanates and isothiocyanates with *N*-arylpyrazole with isolation of intermediate products.

The next two-component procedure with *N*-arylpyrazoles is one of the oldest synthetic pyrazoloquinolines methods (Scheme 38). In 1936, Koćwa published a series of papers in which he obtained 2*H*-pyrazolo[3,4-*b*]quinolines **140** by reacting aryl isocyanates **137** or isothiocyanates **138** with *N*-arylpyrazoles **136** [3,4]. By heating *N*-[5-methyl-1-phenyl-1,2-dihydro-3*H*-pyrazol-3-ylidene]aniline **136** with phenyl isocyanate **137** at a temperature of 230–240 °C, product **140** is formed, while, in the case of phenyl isothiocyanate **138**, intermediate **139** was isolated, which then cyclised to **140** by heating with PCl_5_ at a temperature of 100–110 °C in 15 min (Scheme 38).

**Scheme 39:**
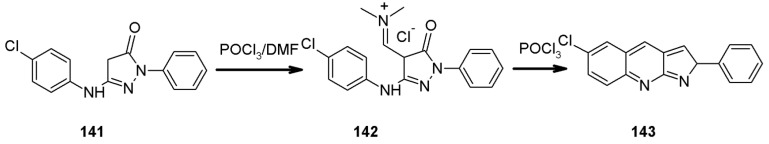
Ring closure by Vilsmeier–Haack formylation reaction.

In 1976, Purnaprajna and Seshadri, as a result of the Vilsmeier–Haack formylation of 1-phenyl-3-(*p*-chloroanilino)-5-pyrazolone **141**, isolated 4-dimethylaminomethylene derivative **142**. In the next stage, they cyclised it by using phosphorus oxychloride to produce 3,6-dichloro-2-phenyl-2*H*-pyrazolo[3,4-*b*]quinoline **143** (Scheme 39) [89].

For a long time, this was only one example of such reaction. It was not until 2021 that Kucharek and colleagues developed a one-step cyclisation reaction of 5-*N*-arylpyrazoles **132** in the presence of dimethylformamide diethyl acetal (DMF-DEA)/POCl_3_ at 80 °C. The yields of the obtained pyrazoloquinolines **59** ranged between 27 and 97% [90].

**Figure 11:**
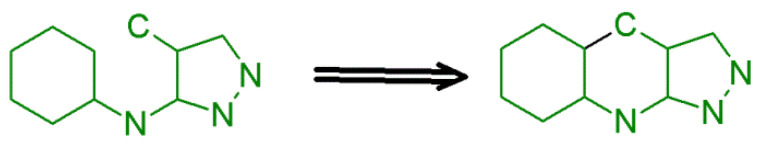
Path 6b: C1_6b_: C4-C4a.

We include in this part one more synthesis, in which the 5-*N*-arylamino derivatives of pyrazoles with the ester group in the 4 position of the pyrazole ring are obtained (Figure 11. Path 6b).

**Scheme 40:**
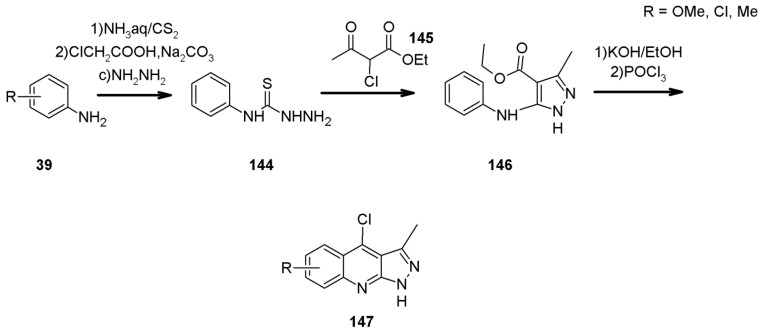
Synthesis of 5-chloro-3-methyl-1*H*-pyrazolo[3,4-*b*]quinoline derivatives.

This compound can be easily prepared in a one-pot reaction by starting from aniline **39**, carbon disulfide, chloroacetic acid and hydrazine, which yields thiosemicarbazide **144**. A subsequent reaction with ethyl 2-chloroacetoacetate **145** produced pyrazole ester **146**. The hydrolysis of **146** in EtOH/KOH and the cyclisation of the resulting acid with POCl_3_ yielded chloro derivative **147** (Scheme 40) [33,91,92,93].

### 2.10. 1H-Pyrazolo[3,4-b]quinoline Syntheses Based on 4-arylidenepyrazoles

**Figure 12:**
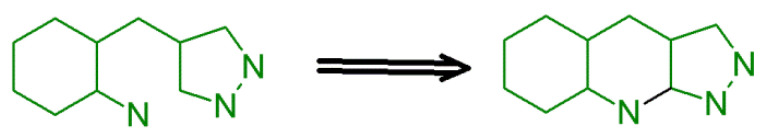
Path 7; C1_7_: N9-C3a.

Another one-component procedure is the synthesis which uses 4 arylidene substituted pyrazole derivatives (Figure 12).

**Scheme 41:**
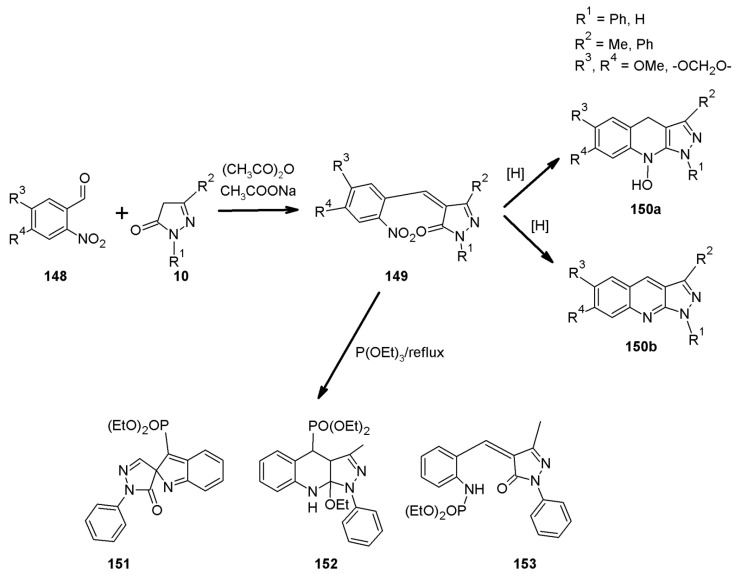
*o*-Nitrobenzaldehyde as an equivalent for *o*-aminobenzaldehyde in the synthesis of pyrazolo[3,4-*b*]quinolines.

Coutts and Edwards reacted *o*-nitrobenzaldehyde **148** and pyrazolone **10**, which yielded 4-(2-nitrobenzylidene)-2-pyrazolin-5-ones **149** [94]. The next step was to use several methods of reductive cyclisation, such as cyclohexene/Pd(C), NaBH_4_ as well as zinc and acetic acid. The end product was 9-hydroxypyrazolo[3,4-*b*]quinolines **150a**. After changing the reaction conditions as a result of reductive cyclisation, they obtained pyrazolo[3,4-*b*]quinoline **150b** [95] (Scheme 41). The prereaction between *o*-nitrobenzaldehyde **148** and the corresponding pyrazolone **10** may be an alternative to avoid the synthesis of the anthranilic aldehyde **1** (R^3^ = H, R^4^ = H, halogen, OMe) that is needed for the Friedländer condensation (Scheme 3).

As part of the research on nitrenes, Kametani and colleagues performed reductive cyclisation reactions on 4-(4,5-methylenedioxy-2-nitrobenzylidene)-2-phenyloxazolones by using triethyl phosphite (P(OEt)_3_). These compounds were prepared from 4,5-dimethoxy-2-nitrobenzaldehyde (**145**; R^3,4^ = OMe) and *N*-benzoylglycine. The reaction resulted in the formation of 2-phenyloxazolo[5,4-*b*]quinoline [96]. Nishiwaki also tried to cyclise 4-(2-nitrobenzylidene)-2-pyrazolin-5-ones **149** by using P(OEt)_3_ as the reducing agent. However, instead of the expected pyrazoloquinolines, a whole range of other products were isolated from the reaction mixture, the structures (e.g., **151, 152**, **153**) of which are shown in Scheme 41 [97]. Tomasik and Danel used reductive cyclisation with iron powder and glacial acetic acid to synthesise pyrazoloquinolines **150** from *o*-nitrobenzylidene derivatives **149**, however with moderate yields [98].

**Scheme 42:**
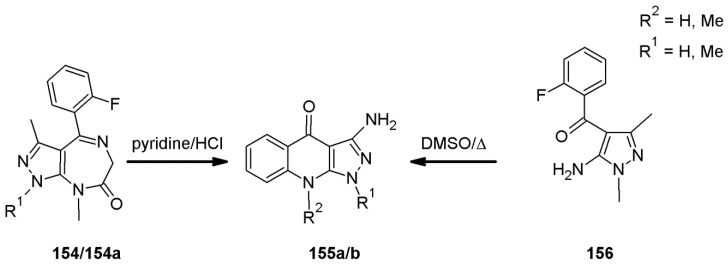
Investigation on Zolazepam metabolites.

In concluding the discussion of the use of single-component systems, one could mention the work of DeWald on the metabolites of Zolazepam **154** (R^1^ = Me). After the administration of Zolazepam to rats, the metabolite **154a** (R^1^ = H) was found in their urine. In order to synthesise it, the Zolazepam was demethylated with boiling pyridine hydrochloride; however, the reaction was unsuccessful, and instead of **154a**, **155a** was obtained (R^2^ = Me, R^1^ = H). Compound **156** was synthesised to obtain an isomer **155b** (R^2^ = H, R^1^ = Me) (Scheme 42) [99].

To sum up, the synthesis of pyrazoloquinolines with the use of a single-component system (e.g., **148**) is of no practical importance in the light of other, much more efficient methods.

### 2.11. Multicomponent 1H-Pyrazolo[3,4-b]quinoline Synthesis

**Figure 13:**
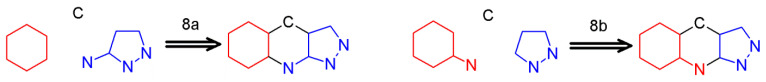
Path 8a; C4-C4a, C4-C3a, C8a-N9. Path 8b; C4-C4a, N9-C9a, C4-C3a.

In recent years, the significant development of multicomponent reactions in organic chemistry has been observed, including the syntheses of heterocyclic compounds [100,101]. Many review publications and monographs on this issue have been published [102,103]. As far as pyrazoloquinolines are concerned, the multicomponent reactions described so far in the literature are limited to the two methodologies presented below (Figure 13).

**Scheme 43:**
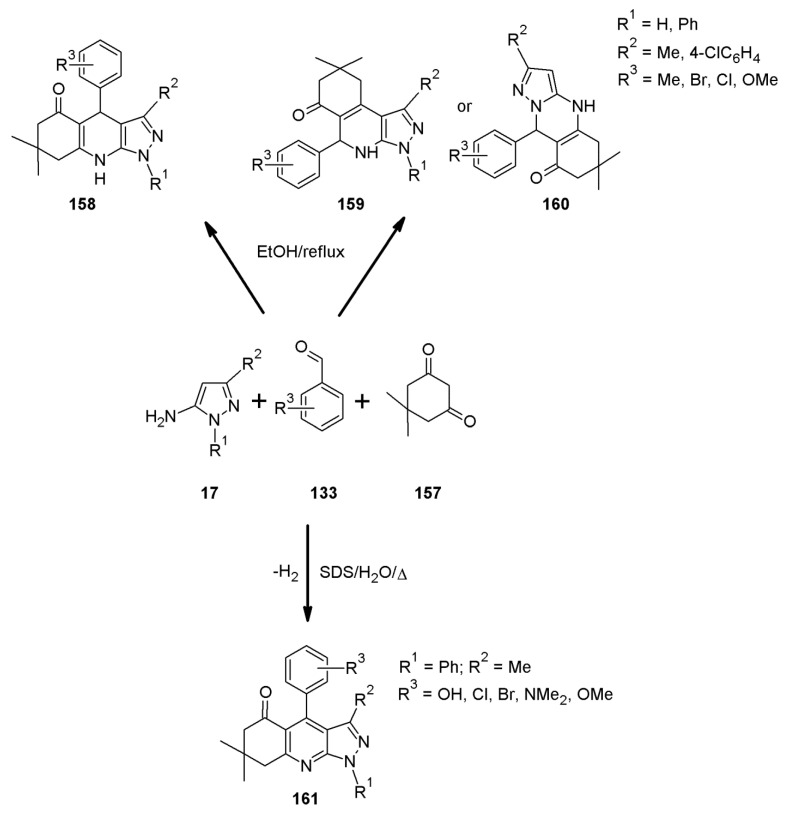
The first three-component synthesis of 4-aryl-4,7,8,9-tetrahydro-6*H*-pyrazolo[3,4-*b*]quinolin-5-ones.

The first reaction of this type was described by Hormaza et al. in 1998. Pyrazoloquinoline derivatives **158** were obtained by heating the amino pyrazole **17**, aromatic aldehyde **138** and the corresponding cyclic 1,3-dione **157.** The authors proved that the reaction is regiospecific with the formation of linear pyrazoloquinoline **158** when R^1^ = H or Ph. The angular derivatives **159** or **160** (for R^1^ = H) were not formed (Scheme 43) [104].

Nogueras et al. investigated the mechanism of the abovementioned reaction. After carrying out a few experiments, they came to the conclusion that the first step is the Knovenagel condensation between dimedone **157** and aldehyde **133**, and then, in the Michael addition, there is the attachment of aminopyrazole **17** with the subsequent cyclocondensation between the dimedone carbonyl group and the pyrazole amino group [105]. This reaction has been modified many times in terms of different reaction conditions. These modifications include conducting reactions in the microwave field, with or without a catalyst such as *L*-proline [106,107]. One of the important synthetic aspects of green chemistry is the use of ultrasound in organic synthesis. This approach was used by Maddila and colleagues for the synthesis of pyrazoloquinolines. The reactions took several minutes, and the yields of the products that were obtained were relatively high (in the order of 70–98%) [108]. Other modifications to this reaction consisted of the use of various catalysts, such as H_3_PW_12_O_14_ or nanomagnetic celulose in ionic liquids [109,110]. In all mentioned cases, when dimedone **157** was used, the central heterocyclic ring was hydrogenated. In some cases, a fully aromatic ring was produced (Scheme 43). Thus, Shi and Wang used sodium 1-dodecanesulfonate SDS as a catalyst in the aqueous reaction medium for the same components (**17**, **133** and **157**) [111]. The end product was a system with a fully aromatic pyridine fragment **161**. The authors mentioned in the paper that the mechanism of this reaction is not fully understood, and especially how the hydrogen molecule is lost from the structure. The same reaction that was carried out in polyethylene glycol PEG-400 yielded the same product with an aromatic pyridine fragment [112].

**Scheme 44:**
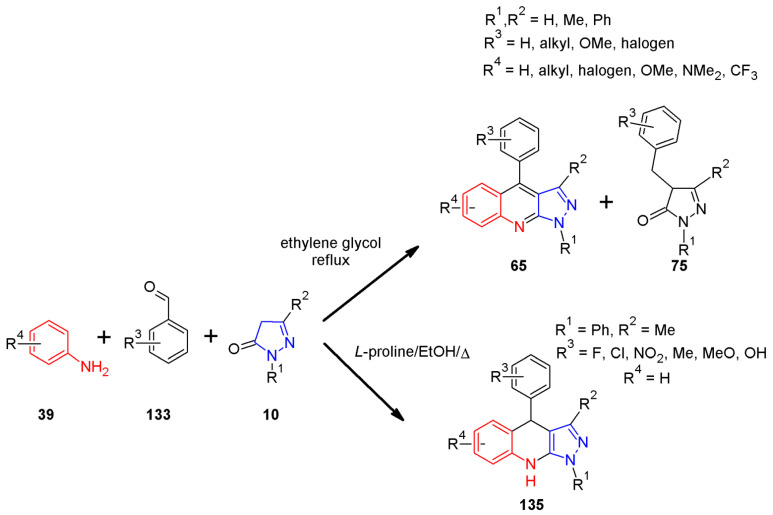
The first multicomponent synthesis of aromatic 1*H*-pyrazolo[3,4-*b*]quinolines.

Contrary to the previously described multicomponent reactions (Path 8a; C4-C4a, C4-C3a, C8a-N9), Tomasik et al. synthesised fully aromatic pyrazolo[3,4-*b*]quinolines **65** (Path 8b; C4-C4a, N9-C9a, C4-C3a) (Scheme 44) [113].

The final compounds **65** are obtained by heating a mixture of aromatic amine **39**, aromatic aldehyde **133** and pyrazolone **10** in ethylene glycol for two hours. After cooling and digestion with methanol/ethanol, the pyrazoloquinolines precipitate as a crystalline solid. The yields of the reactions are in the range of 20–33%, although some authors have claimed that they obtained pyrazoloquinolines **65** in the order of 50–60% by this method [114]. Aromatic amines **39** can contain both electron-donating groups and electron-withdrawing groups in the *o*, *m*, or *para* positions. Aromatic aldehydes **133** can include various benzaldehyde derivatives, as well as naphtalene-1/2-carbaldehyde, but it is unable to obtain derivatives of 9-formylanthracene. Despite moderate yields, it seems to be the best method of obtaining 4 substituted aryl pyrazoloquinolines, which successfully replaces the Friedländer synthesis by using substituted *o*-aminobenzophenones. Hedge and Shetty describe a multicomponent synthesis of 1,4-diphenyl-3-methyl-4,9-dihydro-1*H*-pyrazolo[3,4-*b*]quinolines **135** that used *L*-proline as the catalyst (Scheme 44) [115]. The components were the same as in Tomasik et al.’s procedure, but the final product was not aromatised in the final step (Path 8b; C4-C4a, N9-C9a, C4-C3a).

**Scheme 45:**
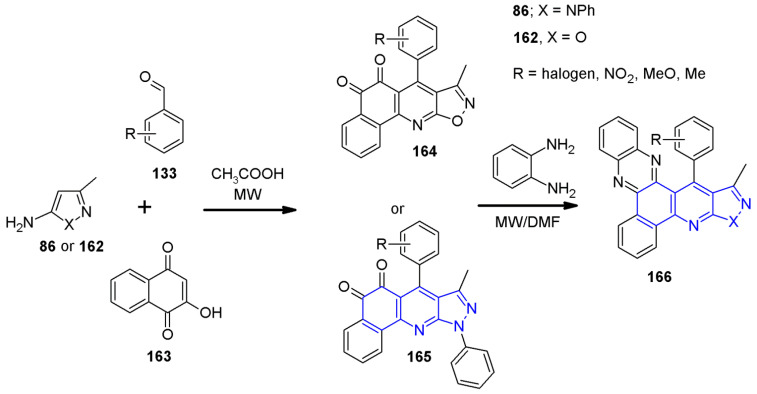
Syntheses of benzo[*h*]pyrazolo[3,4-*b*]quinolin-5,6-diones and derivatives.

Another group of multicomponent reactions are those that use 2-hydroxynaphtalene-1,4-dione **163**. Li et al. synthesised two series of compounds: benzo[*h*]isoxazolo[5,4-*b*]quinolines **164** from 5-amino-3-methyloxazole **162**, and benzo[*h*]pyrazolo[3,4-*b*]quinolines **165** by applying 5-amino-3-methyl-1-phenylpyrazole **86** (Scheme 45) [116].

In the next stage, the authors subjected the obtained systems (**164** and **165**) to reactions with 1,2-diaminobenzene in order to obtain the quinoxaline derivatives **166**. All the steps that are described were carried out under microwave irradiation.

The reaction that was performed by Rajesh et al. is described as a “*sequential four-component reaction*”, which makes it a kind of record holder among the multicomponent reactions that are used for the synthesis of pyrazoloquinolines **165**. They reacted 3-aminocrotononitrile and phenylhydrazine with an addition of *L*-proline for 10 min in boiling water, and they then added an equimolar mixture of aldehyde **138** and dione **163**. The whole mixture was then heated for 1–1.5 h [117].

Quiroga et al. synthesised a series of benzo[*h*]pyrazolo[3,4-*b*]quinolines **165** via a three-component reaction under microwave irradiation [118]. When they used **86** (X = NH), the middle heterocyclic ring was unsaturated. The formation of the linear benzo[*g*]pyrazolo[3,4-*b*]quinoline system was not observed. The resulting compounds were screened against some Mycobacterium strains.

**Scheme 46:**
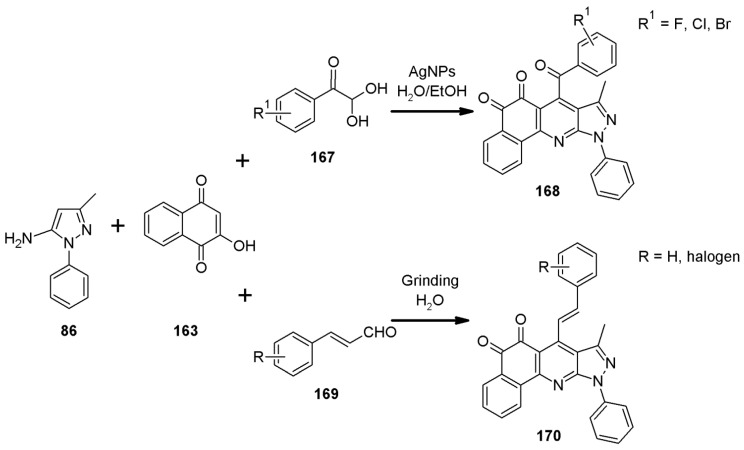
Syntheses of benzo[*h*]pyrazolo[3,4-*b*]quinolin-5,6-diones.

Khalafy et al. conducted research on the influence of silver nanoparticles (AgNPs) on the course of the reaction. The aryl glyoxal hydrates **167** were used as the C-4 carbon incorporation reagent in the three-component reaction. The end product is 7-benzoyl-benzo[*h*]pyrazolo[3,4-*b*]quinolin-5,6-diones **168**. It should be emphasised that the reactions were carried out in a water–alcohol environment at a temperature of 60 °C, and in most cases, they were completed within 60 min [119] (Scheme 46).

Another multicomponent reaction for the synthesis of pyrazoloquinoline derivatives **170** differs from the others by the procedure in which ingredients such as aminopyrazole **86**, 2-hydroxy-1,4-napthalenedione **163** and cinnamaldehyde **169** are ground in a mortar with a little addition of water. The authors call it, “*liquid assisted grinding or LAG*.” After grinding is completed, the product is simply recrystallised from an appropriate solvent (Scheme 46) [120].

The multicomponent reactions with the use of **163** that have been described so far led to the preparation of angular benzo[*g*]pyrazolo[3,4-*b*]quinolin-5,6-diones (e.g., **165**, **168** or **169**). It is also possible to obtain a linear system of the mentioned heterocyclic system.

**Scheme 47:**
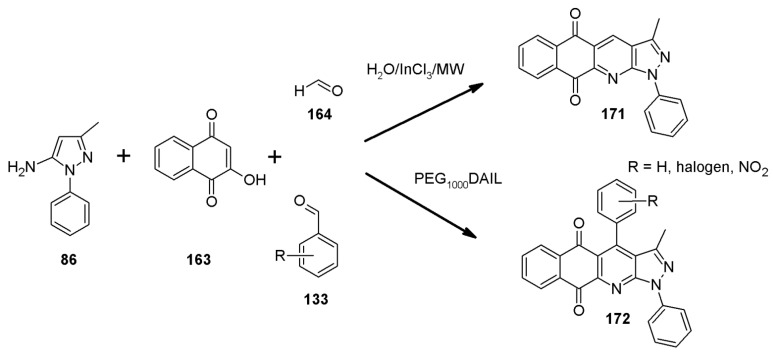
Three-component reactions catalysed by InCl_3_ in synthesis of linear pyrazolo[3,4-*b*]quinoline skeleton.

Gutierez, in the three-component reaction of aminopyrazole **86**, 2-hydroxy-1,4-napthalenedione **163** and formaldehyde **164**, and indium chloride (InCl_3_) as a catalyst, obtained 3-methyl-1-phenylnaphtalen [2,3-*e*]pyrazolo[3,4-*b*]pyridine-5,10-dione **171** (Scheme 47) [121]. The reactions were carried out by using either conventional heating (40–60 h) or by heating in a microwave field (10–20 min).

Indium chloride (InCl_3_), as a catalyst, was also used in the three-component reaction to synthesise not only pyrazoloquinolines, but also pyrimidine derivatives. Instead of formaldehyde, the authors used a number of aromatic aldehydes. In the case of 1,3-cyclohexanedione, a product with a hydrogenated pyridine ring was obtained, and, in the case of 1,3-pentanedione, indane-1,3-dione and naphthalene-2-hydroxy-1,4-dione, which are products with aromatic pyridine fragments, were obtained [122]. Wu et al., as a catalyst, used a 10–15 mol% amount of (NH_4_)_2_HPO_4_ for 2-hydroxynaphthalene-1,4-dione **163**, aromatic aldehyde **133** and 5-amino-3-methyl-1-phenylpyrazole **86** three-component reactions [123]. The isolated yields of the benzo[*h*]pyrazolo[3,4-*b*]quinolines ranged from 80–95%.

Due to the concern for the protection of the natural environment, we have observed an increasing tendency to change the approach to organic synthesis. This is manifested, inter alia, by the use of water as the reaction medium, or by the use of ionic liquids. One such example is PEG_1000_-based dicationic acid ionic liquid, which has been used by Ren et al. in the synthesis of linear benzo[*h*]pyrazolo[3,4-*b*]quinolines **172** in three-component reactions from **163, 133** and **86** [124].The authors presented a possible mechanism of this reaction that consists of the addition of an aldehyde **133** to **163**, followed by a Michael’s addition of aminopyrazole **86**, the cyclisation of the resulting adduct with the subsequent oxidation with air and the final formation of **172** (Scheme 47).

*L*-proline is a frequently used catalyst in multicomponent reactions. Karamthulla et al. used it in the synthesis of linear 2*H*-benzo[*g*]pyrazolo[3,4-*b*]quinolone-5,10(4*H*,11*H*)-dione derivatives [125]. The reaction that was performed differed slightly in terms of the choice of components, and, namely, by the fact that 3-amino-5-methylpyrazole was used instead of the 1-phenyl-3-methyl-5-aminopyrazole **86**. Contrary to the reactions that are shown in Scheme 47, no aromatisation of the pyridine ring was observed.

**Scheme 48:**
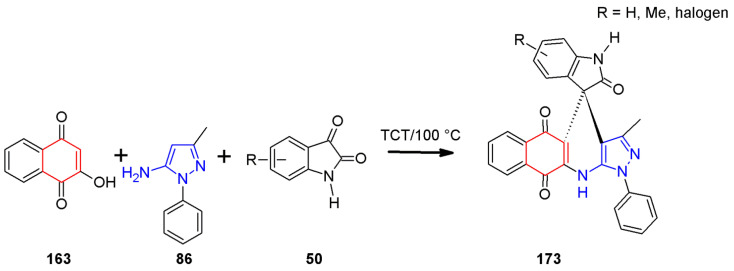
Synthesis of spiro[benzo[*h*]pyrazolo[3,4-*b*]quinoline-4,3′-indoline.

In multicomponent reactions where pyrazoloquinolines are synthesised, isatin is sometimes used. We mentioned it in the context of the Pfitzinger reaction that was described earlier. In this case, isatin made a significant contribution to the construction of the pyrazoloquinolines skeleton (Scheme 17). In the three-component reactions that are depicted in Scheme 48, it contributes only carbon 4 in the skeleton (Path 8a; C4-C4a, C4-C3a, C8a-N9). Wu et al. obtained spiro[benzo[*h*]pyrazolo[3,4-*b*]quinoline-4,3′-indoline] **173** by reacting dione **163**, aminopyrazole **86** and isatin, or its derivative **50**, in the presence of wet cyanuric chloride (TCT) [126].

Dabiri conducted the abovementioned reaction in the presence of *L*-proline (10 mol%) in boiling water for 6–7.5 h. The researchers additionally conducted additional studies by replacing isatin with acenaphtylen-1,2-dione. The yields of the corresponding spiro derivatives were in the range of 50–70% [127]. Spiro compounds based on pyrazoloquinolines (i.e., spiro[indoline-3,4’-pyrazolo[3,4-*b*]quinoline]-2.5’(6’*H*) dione) can also be obtained without any catalysts, as was proven by Rong and his colleagues by heating isatin **50**, dimedone **157** (or 1,3-cyclohexanedione **43**) and 3-amino-1*H*-pyrazole in water or dilute acetic acid [128]. This reaction meets 100% of the requirements of the so-called “green chemistry”.

The catalysts that have been discussed so far in multicomponent reactions were of a homogeneous nature. Baradani and colleagues used Fe_3_O_4_@Cu(OH)x as a catalyst in the three-component reaction of dimedone **173**, aminopyrazole **86** and 5,6-dihydro-4*H*-pyrrolo[3,2,1-*ij*]quinoline-1,2-dione, instead of isatin [129]. The reactions were carried out in the environment of water/ethanol with the addition of a nanocatalyst, and they lasted from 8 to 9 h. After the reaction was completed, the catalyst was removed with a strong magnet. The final product was separated and purified with chromatographic techniques.

**Scheme 49:**
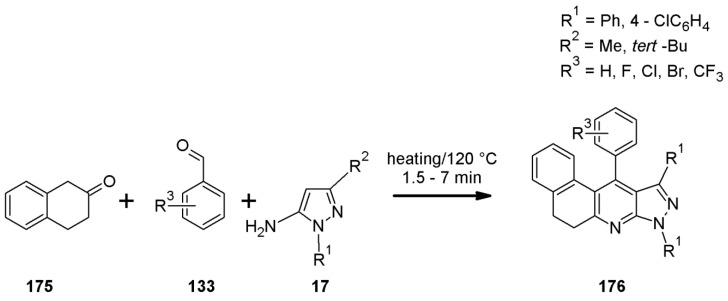
*β*-Tetralone as a substrate for benzo[*f*]pyrazolo[3,4-*b*]quinoline synthesis.

In the literature, you can find descriptions of three-comonent reactions that use *β*-tetralone **175** or *α*-teralone **177** for the synthesis of angular benzo[*f*]-**197** or benzo[*g*] pyrazoloquinolines **200**. The reactions were carried out by melting the reagents [130]. The first of the abovementioned reactions ran smoothly and produced the expected benzo[*f*] derivative **176** (Scheme 49).

**Scheme 50:**
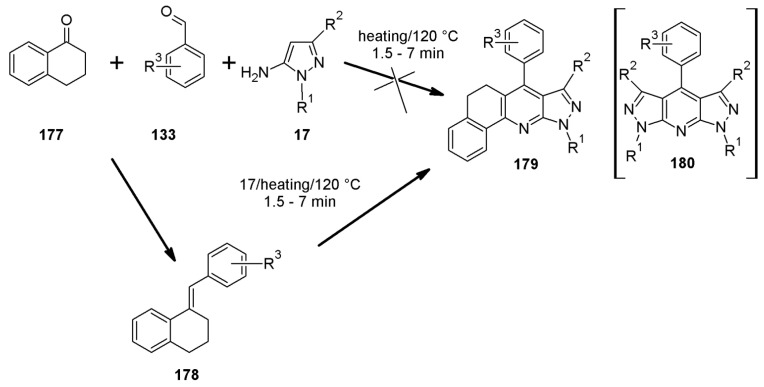
*α*-Tetralone as a substrate for benzo[*h*]pyrazolo[3,4-*b*]quinoline synthesis.

Quiroga and co-workers tried to repeat the earlier procedure to obtain the benzo[*h*]isomer **179**, and unfortunately, in this case, they were not successful. The main product turned out to be *bis*-pyrazolo[3,4-*b*;3’,4’-*e*]pyridine **180**. Therefore, they changed the procedure and first synthesised a 2-arylidene derivative of *α*-tetralone **178**, which was then reacted with aminopyrazole **17** by melting. This time, a derivative **179** was obtained (Scheme 50).

**Scheme 51:**
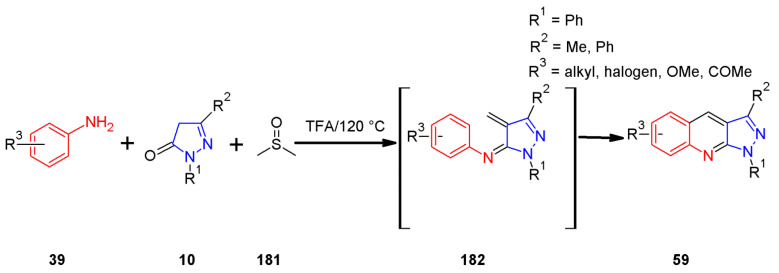
Three-component reaction with DMSO as source of C-4 atom in pyrazolo[3,4-*b*]quinoline skeleton.

Sathiyanarayanan et al. prepared benzo[*f*]pyrazolo[3,4-*b*]quinolines **176** by heating *β*-tetralone **175**, aminopyrazole **17** and aromatic aldehyde **133** in ethanol/CH_3_COOH and tin chloride (SnCl_2_) (10 mol%) [131]. The reaction also proceeded with cyclohexanone and cyclopentanone, but it failed with α-tetralone and 2-hydroxynaphtalene-1,4-dione **163**. The resulting compounds exhibited intense emission properties in solution, and in the solid state as well. Moreover, when the R^3^ was NMe_2_, the compound exhibited aggregation-induced emission AIE. To conclude our review of the most important pyrazoloquinoline reactions in the last 100 years, we were very pleased with the work that was published by Tiwari et al. in 2021 [132]. It is a very universal method that allows one to obtain a whole range of pyrazoloquinolines **59** from commercial ingredients, such as aromatic amines **39**, pyrazolones **10** and dimethylsulfoxide **181**. The reaction proceeds in the presence of trifluoacetic anhydride TFA. The authors proposed the mechanism of this reaction, where one of the intermediate steps is the azadiene system **182**, which is annulated and then aromatised to produce the final pyrazolo[3,4-*b*]quinoline **59** (Scheme 51).

Perhaps this reaction will be an alternative to the procedure that was developed by Brack in 1965 (Scheme 19), which allows the obtainment of all of the possible combinations of the substituents for **10** (R^1^, R^2^ = H, Me, Ph) in the pyrazole ring. Tiwari et al. employed only two pyrazolone derivatives **10** (R^1^ = Ph, R^2^ = Me and R^1,2^ = Ph). Finally, it may be mentioned that the authors expected a completely different result; thus, this is an example of serendipity in organic chemistry.

## 3. Photophysical Physical Properties of 1*H*-Pyrazolo[3,4-*b*]quinolines and Their Application

In view of the ever-growing demand for many high-tech and biomedical applications, the group of 1*H*-pyrazolo[3,4-*b*]quinolines has gained considerable interest from the scientific community, which is mainly due to their intrinsic optical and photophysical characteristics. Over the past decades, many researchers have made some efforts to understand the relationship between the structure and the optical properties of these compounds. However, even though the establishment of the structure obtained by Niementowski was made in 1928 [2], it was only in the late 1990s and mid 2000s that a number of reports related to the spectroscopic characteristics and the computational analysis of 1*H*-pyrazolo[3,4-*b*]quinoline derivatives (see Figure 1 in the Introduction) were published [133,134,135,136,137,138].

### 3.1. Structure–Property Relationship

**Figure 14:**
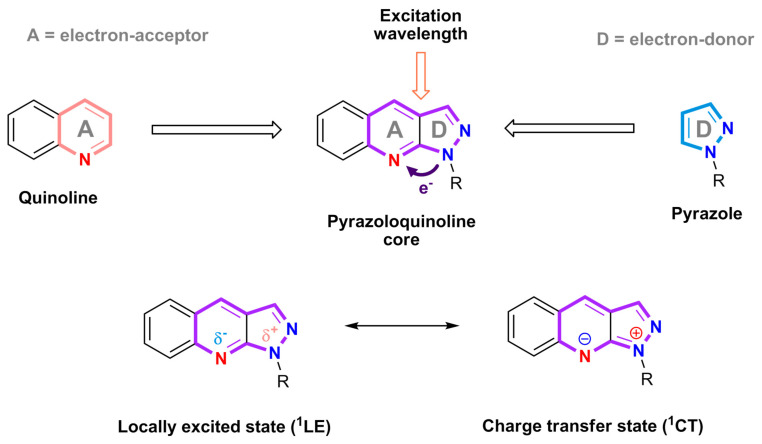
General structure and the light-induced formation of brightly emissive D-π-A pyrazoloquinoline species.

Structurally, pyrazoloquinolines are the products of the ring fusion of the heteroaromatic pyrazole and quinoline moieties, which gives rise to a rigid D-π-A system (Figure 14). Separately, these building blocks play a crucial role in the design of many functional materials. For instance, quinoline scaffolds are frequently used as electron-accepting components in light-harvesting systems [139,140], or as optical limiters [141]. Furthermore, quinoline-based molecules exhibit highly efficient electron-transporting properties that are combined with thermal and redox robustness and high photoluminescence quantum yields. These features are of paramount importance in the construction of many high-tech devices, such as organic optoelectronics (OLEDs) [142,143], photodiode detectors [144] and photovoltaic cells (OPVs) [145]. On the other hand, the pyrazole unit often acts as an electron donor in chromophore systems, which is due to the presence of two electron-rich adjacent nitrogen atoms [146,147]. Especially in conjunction with various electron acceptors (e.g., electron-deficient aromatics and heteroaromatics), pyrazole derivatives emerge as highly efficient, tunable light emitters [148,149,150]. Such structural motifs, including pyrazoloquinolines, have been intensively studied in the area of advanced dye chemistry, and most recently as electroluminescent materials [6,151,152], fluorescence sensors [153,154] and second-order nonlinear optical materials [155,156,157].

**Figure 15:**
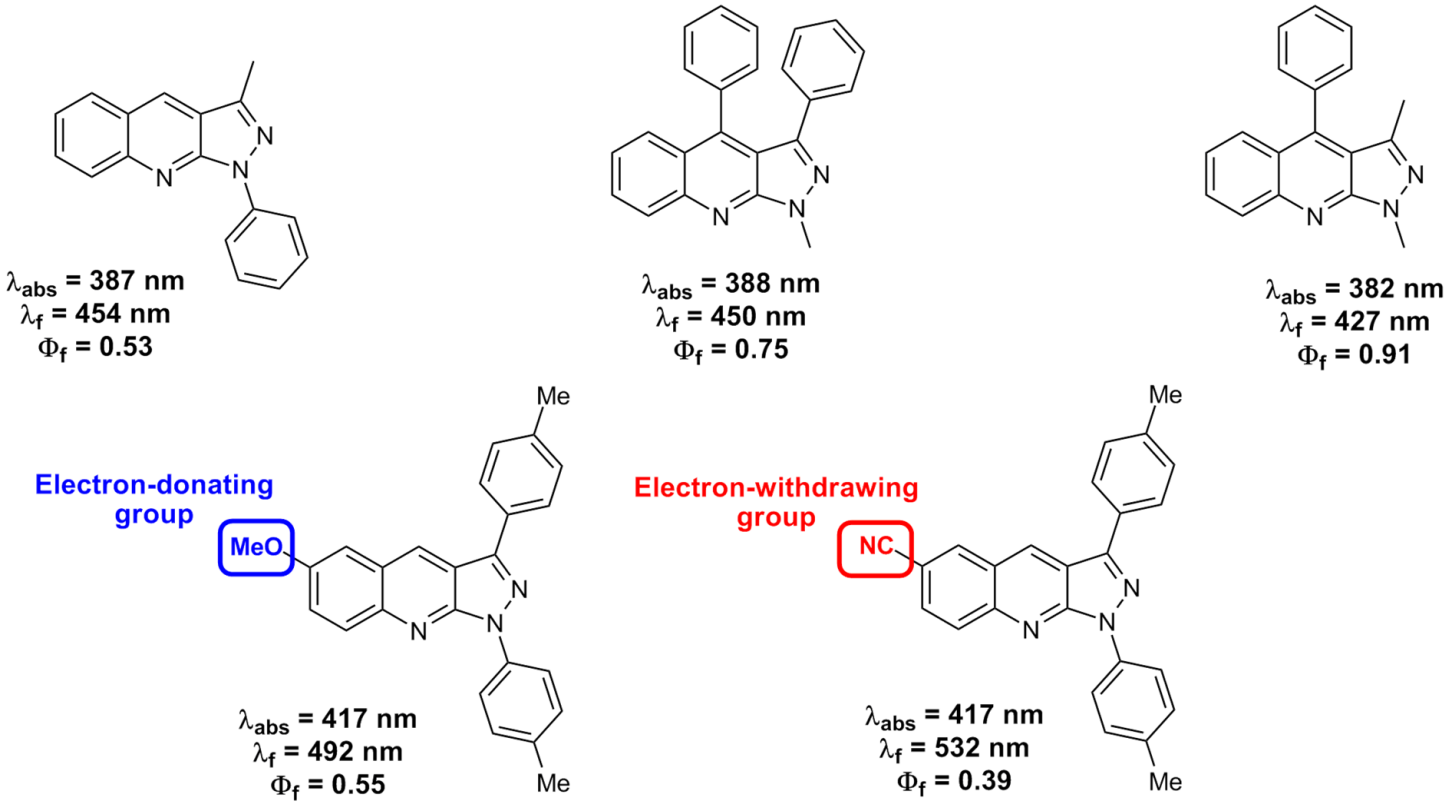
Structures and photophysical properties of differently substituted 1*H*-pyrazolo[3,4-*b*]quinolines. The data presented were measured in chloroform [158].

Generally, the absorption spectra of 1*H*-pyrazolo[3,4-*b*]quinolines contain two absorption bands in the UV–Vis region of light: I) The broad S_0_→S_1_ (π→π*) transition with an absorption maxima that ranges from ca. 380 to 420 nm; and II) More or less pronounced (depending on the substitution pattern) multiple bands at shorter wavelengths (<300 nm), which can be attributed to S_0_→S_n_ (mixed π→π* and n→π*) transitions. Furthermore, it can be observed that, in the case of pyrazoloquinolines substituted with donor and/or acceptor groups, the long-wavelength absorption maxima are slightly redshifted, corresponding to their unsubstituted analogues [47,158,159]. In fact, light-induced transitions in D-π-A chromophores are directed from an electron-rich to an electron-deficient unit, which results in a significant charge separation within the excited state (see Figure 14) [160]. In most cases, such a charge separation leads to the formation of conformationally disrupted and poorly emissive intramolecular charge transfer (ICT) states. However, the rigidity of the pyrazoloquinoline skeleton promotes electronically allowed emission, as the frontier orbitals that participate in the ^1^CT→S_0_ transition have parallel orientation and are, therefore, markedly overlapped [161]. Subsequently, 1*H*-pyrazolo[3,4-*b*]quinolines are highly fluorescent in the blue or greenish-blue parts of the spectrum, with quantum fluorescence yields reaching unity in some cases (Figure 15) [20,158,162].

A tremendous variety of synthetic approaches has led to an extension of the portfolio of pyrazoloquinolines with tunable luminescence features. Exemplarily, an introduction of additional electron-donating and electron-withdrawing groups within the chromophore results in the formation of spatially extended dipolar (or quadrupolar) systems. A considerable charge separation within the electronically excited states of these molecules results in a redshift of the emission bands, which noticeably proceeds with an increase in the solvent polarity [138,159,163]. However, the fluorescence intensity of such organic dyes decreases at the same time, which is due to an enhancement of the rate of nonradiative decay via intramolecular rotations within excited ICT states [164,165]. Another significant process that leads to the rapid deactivation of excited states is referred to as “photoinduced electron transfer (PET)” [166,167,168,169]. In this case, an electron donor (e.g., dialkylamine group) and the luminophore are commonly separated by a short alkyl spacer, which electronically disconnects the π-conjugation between the receptor and the electron donor units. The excitation of such a system is followed by the PET process, which leads to an immediate nonradiative decay to the ground state. However, most of the pyrazoloquinolines that are substituted with dialkyl and diaryl amines are deliberately designed for the in-active interruption of the aforementioned fluorescence-quenching mechanisms, which makes them promising “turn-on” sensors.

### 3.2. Application of Pyrazoloquinolines in Fluorescence Sensing

**Figure 16:**
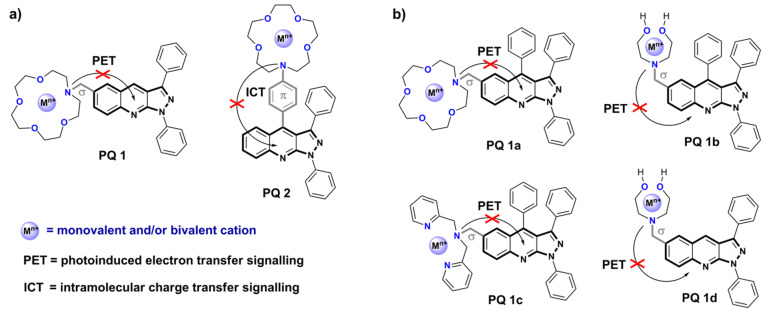
Structures of pyrazoloquinoline-based cation-sensitive fluorescence probes: (**a**) aza-crown-modified derivatives, reported by Rurack in 2002 [5]; (**b**) PET-signalling derivatives, investigated by Mac et al. between 2010 and 2013 [175,176,177].

Since the electroluminescence properties of 1*H*-pyrazolo[3,4-*b*]quinolines have been frequently reported for many years [20,47,170,171,172,173,174], in this review, we focus on their use as efficient fluorescence probes. The operation of many fluorescent indicators is based on a noticeable enhancement of the fluorescence emission upon the addition of metal cations to the fluorescing medium. Exemplarily, in 2002, Rurack et al. reported the synthesis of two aza-crown-modified 1,3-diphenyl-pyrazoloquinolines that exhibited high fluorometric sensitivity to Na^+^ and Ca^2+^ cations (see Figure 16a) [5]. The main concept of this study relied upon the structural connection between the nitrogen lone pair of the analyte receptor and the chromophore system, either by a σ-spacer (compound **PQ 1**) or by a perpendicular π-conjugated arrangement (compound **PQ 2**). Derivative **PQ 1** proved to be weakly emissive in polar solvents (Φ_f_ = 0.02 in acetonitrile, compared to Φ_f_ = 0.54 in hexane), which was mainly due to the PET-fluorescence-quenching mechanism. On the contrary, pyrazoloquinoline **PQ 2** showed an intense dual emission from LE and ICT fluorescent species (Φ_f_ = 0.18 in acetonitrile, compared to Φ_f_ = 0.37 in hexane). Furthermore, both derivatives showed highly desirable effects in the presence of analyte species (i.e., metal ions) on the basis of two different sensing mechanisms. The authors demonstrated that dye **PQ 1** readily bound monovalent Na^+^ and bivalent Ca^2+^ cations, with a concomitant increase in the fluorescence intensity. These observations were in unambiguous agreement with a proposed PET-signalling mechanism. More interestingly, pyrazoloquinoline **PQ 2** performed as a dual emissive sensor with a high fluorescence output for both states of the detection system: bound and unbound. In this case, the presence of bivalent Ca^2+^ cations alternated the nature of the excited state of the fluorophore from the low-lying ICT state to the blue-shifted LE state, with a significant enhancement in the emission (from Φ_f_ = 0.18 to Φ_f_ = 0.35 in acetonitrile) at the same time. In contrast, the addition of Na^+^ ions to **PQ 2** did not change its spectral characteristics markedly.

Since the aforementioned studies on pyrazoloquinoline-based sensors were reported, almost a decade passed until this stem was followed. Then, between 2010 and 2013, Mac and co-workers published a series of articles on ion-sensitive pyrazoloquinolines, which varied by the molecular architecture of the receptor unit (see Figure 16b) [175,176,177]. All of the sensors presented (**PQ 1a**–**d**) were designed for PET signalling, and they maintained the connection between the receptor unit and the pyrazoloquinoline chromophore via the non-conjugated methylene spacer. In addition, the authors extended their studies to a wide range of metal cations, including monovalent Li^+^, Na^+^ and Ag^+^, and bivalent Ca^2+^, Ba^2+^, Mg^2+^, Zn^2+^, Cd^2+^ and Pb^2+^ cations. The results obtained were consistent, which showed that these “turn-on” fluorescent cation indicators were more sensitive to the presence of bivalent cations than to monovalent species. Interestingly, for compound **PQ 1c**, an increased sensitivity and selectivity to Zn^2+^ and Mg^2+^ arose from a predominant complexation of these cations with two molecules of the sensing system. Furthermore, noticeable bathochromic shifts of the fluorescence bands were observed for M_2_Zn^2+^ and M_2_Mg^2+^ complexes. These findings were explained by the formation of excimer species [M^2+^(MM)]*, which was confirmed by quantum chemical calculations [177]. In 2013, the same research group demonstrated a detailed study on the pyrazoloquinoline derivative **PQ 1d**, which was a direct precursor for the synthesis of the aza-crown fluorescence probe **PQ 1**, previously reported by Rurack [5]. It was found that this compound can act as a highly efficient sensor for many bivalent cations and that, more importantly, its selectivity can be easily enhanced by the addition of small amounts of water to the fluorescing medium.

**Figure 17:**
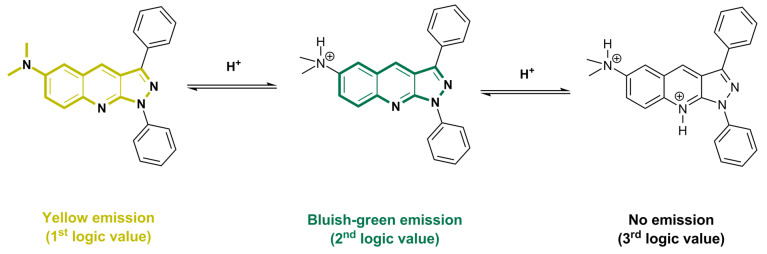
Schematic representation of 1*H*-pyrazolo[3,4-*b*]quinoline-based pH-sensitive ternary logic gate reported by Uchacz et al. in 2016 [178].

Three years later, Uchacz et al. investigated a series of donor-acceptor 1*H*-pyrazolo[3,4-*b*]quinolines that were substituted with different amine donors (i.e., *N*,*N*-dimethylamine, *N*,*N*-diphenylamine, *N*,*N*-phenyl-1-naphthylamine and carbazole groups), with the aim of implementing them as pH-sensitive molecular logic switches [178]. The authors showed that, in the presence of trifluoroacetic acid (the input signal), the fluorescence of the investigated pyrazoloquinolines was almost completely quenched, which was due to the formation of a nonemissive protonated adduct. Interestingly, pyrazoloquinoline substituted with the *N*,*N*-dimethylamine group presented a more advanced ternary logic behavior (see Figure 17). The nonprotonated state of this compound showed considerable fluorescence (yellow emission, first logic value), which, upon the first protonation of the nitrogen of the dimethylamino moiety, shifted hypsochromically and slightly decreased (bluish-green emission, second logic value). The second protonation, which involved the nitrogen of the quinoline core, quenched the fluorescence quantitatively (no emission, third logic value). Basically, reading the fluorescence response as an output that is dependent on the presence of proton input signals yielded functional luminescent molecules with the potential for multilevel logic switching, which ranged from binary to ternary responses.

With regard to all of these results, it can be concluded that the scope of pyrazoloquinoline-based fluorescence probes is still amenable to further investigation. For instance, the up-to-date studies were mainly focused on the PET-signalling mechanism, with only minute mention of the intramolecular rotation-dependent quenching of the excited ICT states. Currently, a plethora of photophysical studies are devoted to many different ICT state-related phenomena, such as TICT, PICT, PPT, TADF, umpolung-ICT and so forth [179,180,181,182,183], which can be used as output signals for analyte compounds. Furthermore, the presented studies focus mostly on inorganic ion-sensing, which leaves future prospects for the many biomedical applications of pyrazoloquinoline fluorophores, such as fluorescence bioimaging, photosensitized diagnoses or therapies.

## 4. Biological Properties of 1*H*-Pyrazolo[3,4-*b*]quinolines

Nitrogen-heterocycles are the most common heterocyclic compounds that occur in living organisms [184], and they are also very often tested as compounds that show biological activity [185,186]. Thus, it is not unusual that pyrazoloquinoline derivatives have attracted the attention of researchers who deal with the biological activity of organic compounds. It is advisable to divide pyrazoloquinoline derivatives into groups according to the kind of biological activity that they possess.

### 4.1. Hypolipemic and Hypocholesteremic Activity

**Figure 18:**
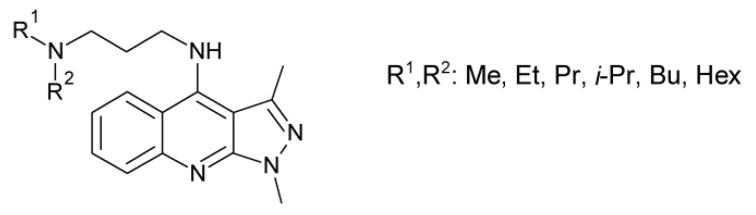
4-[(3-(R^1^,R^2^-Amino)propyloamino]-1,3-dimethyl-1*H*-pyrazolo[3,4-*b*]quinolines.

The method of synthesis that was developed by Stein et al. and Crenshaw et al. [29,30] produced, inter alia, 4-chloro-1,3-dimethyl-1*H*-pyrazolo[3,4-*b*]quinolines, which, after functionalization with different amine derivatives (Figure 18), were tested for their hypolipemic and hypocholesteremic activity in rats [187].

The compounds were suspended in a carboxymethyl cellulose solution at a dose of 100–400 mg/kg, compared to a carboxymethyl cellulose solution only, and were administrated to rats daily for 4 days. After the administration, the rats were tested for their cholesterol and phospholipid concentrations in serum, and the results show that, with the maximum dose of 400 mg/kg, the concentration was reduced significantly: by −51% for cholesterol and by −47% for phospholipids.

### 4.2. Interferon-Production-Inducing Activity

**Figure 19:**
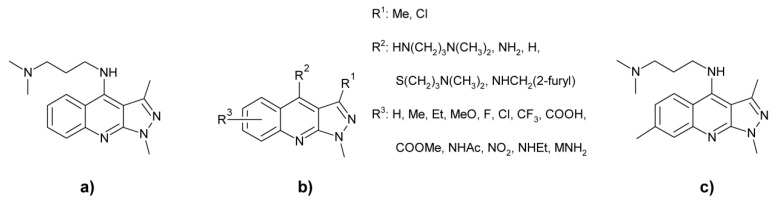
(**a**) 4-[(3-(dimethylamino)propylamino]-1,3-dimethyl-1*H*-pyrazolo[3,4-*b*]quinoline; (**b**) 4-R^2^-1-methyl-3-R^1^-(5,6,7 or 8)-R^3^-1*H*-pyrazolo[3,4-*b*]quinoline; and (**c**) 4-[(3-(dimethylamino)propyloamino]-1,3,7-trimethyl-1*H*-pyrazolo[3,4-*b*]quinoline.

Interferons are an important group of signalling proteins that are released by cells in response to some viruses [188], and interferon-inducing drugs are one of the weapons in the fight against viruses [189]. 4-[(3-(Dimethyloamino)propyloamino]-1,3-dimethyl-1*H*-pyrazolo[3,4-*b*]quinoline (Figure 19), which was obtained by Stein et al. [29], was tested as a low-molecular-weight interferon inducer by Siminoff et al. [190].

The compound was administrated to female mice by the oral or parenteral route, and the administration was repeated. In concentrations of 200 mg/kg or higher, the response of the interferon production was very high, and it protected the mouse L cells against infection by the vesicular stomatotitis virus or the mouse picornavirus. The animals also became hyperesponsive to repeated stimulation. The following research in the Siminoff group also showed that other derivatives of 1,3-dimethyl-1*H*-pyrazolo[3,4-*b*]quinoline, which contained the substituent at the 4 position, which is attached by the NH moiety, and with a side chain of at least three carbons and terminating with the second amino function (Figure 19b), also have significant interferon-inducing activity [30]. In the subsequent research, Siminoff analyzed which cells in mice are the major target for interferon induction by 4-[(3-(dimethyloamino)propyloamino]-1,3-dimethyl-1*H*-pyrazolo[3,4-*b*]quinoline (Figure 19a) [191]. The analysis of the results shows that the type of adherent leukocytes that are resident in the spleen is a major target of interferon induction. The other derivatives (Figure 19b) were also tested by Siminoff and Crenshaw in the cultures of spleen adherent leukocytes [192]. The results show that some of the derivatives also showed high interferon-inducing activity. One of the derivatives, 4-[(3-(dimethyloamino)propyloamino] -1,3,7-trimethyl-1*H*-pyrazolo[3,4-*b*]quinoline hydrochloride (Figure 19c), was also tested by Kern et al. [193], and again, the compound induced high levels of circulating interferon, which effected, with significantly reduced mortality, the mice that were infected with the Rochester mouse virus, *Herpesvirus hominis*, Semliki forest virus and the vesicular stomatitis virus. The authors also observed the strong hyporeactivity of the interferon after multiple doses of 4-[(3-(dimethyloamino)propyloamino]-1,3,7-trimethyl-1*H*-pyrazolo[3,4-*b*] quinoline hydrochloride.

### 4.3. Antiviral Activity

**Figure 20:**
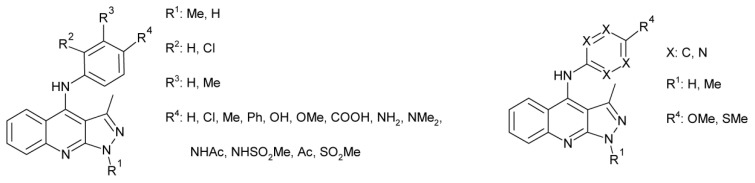
4-(substituted-phenylamino)-1-R^1^-3-methyl-1*H*-pyrazolo[3,4-*b*]quinolines and 4-(4-substituted-pyrimidine-amino)-1-R^1^-3-methyl-1*H*-pyrazolo[3,4-*b*]quinolines.

Many virus infections in humans are self-cured by the immune system of the organism, and, for many others, vaccinations provide immunity to infection. However, there are some diseases that must be cured by the use of antivirial drugs (e.g., HIV, herpes viruses, hepatitis). A huge family of 1*H*-pyrazolo[3,4-*b*]quinoline derivatves (Figure 20), which were obtained by the method of Stein et al. [29] and then functionalised, were synthesised and tested for antiviral activity by the group from the Research Institute for Pharmacy and Biochemistry in Prague in the 1980s [194,195,196,197,198,199,200,201].

**Figure 21:**
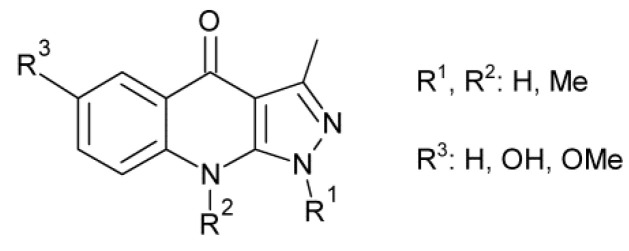
4,9-dihydro-3-methyl-4-oxo-1*H*(2*H*)-pyrazolo[3,4-*b*]quinolines.

Anilino derivatives were tested against the A2-Hongkong virus and against the encephalomyocarditidis (EMC) virus in vivo in mice. Some of them showed high activity against one or the other virus, and some of them were effective against both. In the case of 4-[(4-methylphenyl)amino]- and 4-[(5-methoxy-2-pyrimidinum)amino]- derivatives, which were administrated orally, the survival time was extended after infection with the A2-Hongkong virus by 50 and 67 days, respectively. In the case of 4-[(4-hydroxyphenyl)amino]-, 4-[(4-octadecaoxyphenyl)amino]- and 4-[(3,4-methylenedioxyphenyl)amino]- derivatives that were administrated subcutaneously, the time of survival against the EMC virus was increased by 70, 83 and 95 days, respectively [194,198]. The keto derivatives (Figure 21) were tested against the same viruses as aniline-derivatives: against the A2-Hongkong virus and against the encephalomyocarditidis (EMC) virus in vivo in mice, and after oral or subcutaneous administration.

**Figure 22:**
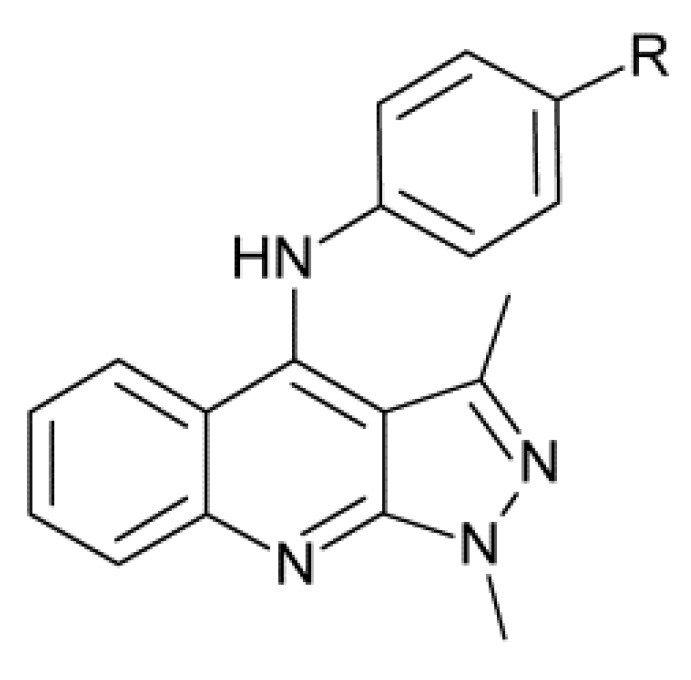
4-[(4-R-phenyl)amino]-1,3-dimethyl-1*H*-pyrazolo[3,4-*b*]quinoline.

The most potent against the A2-Hongong virus was 4,9-dihydro-3,9-dimethyl-4-oxo-1*H*(2*H*)-pyrazolo[3,4-*b*]quinoline, which extended the survival by 55%. In the case of the EMC virus, the compounds were much more effective, and for 4,9-dihydro-3-methyl-4-oxo-1*H*(2*H*)-pyrazolo[3,4-*b*]quinoline and 4,9-dihydro-6-methoxy-3-methyl-4-oxo-1*H*(2*H*)-pyrazolo[3,4-*b*]quinoline, the survival was extended by 96% or by 80–100%, respectively [195,200,201]. Some of the compounds were also tested in vitro against different microbes (e.g., *Streptococcus faecalis*, *Escherichia coli* or *Candida albicans*); however, none of the studied derivatives had significant inhibitory effects [195]. The authors from the Research Institute for Pharmacy and Biochemistry in Prague were so satisfied with the results that they obtained for some of the tested pyrazoloquinoline derivatives that they patented them in Czechoslovakia [202,203,204,205,206,207,208]. One of the compounds that was obtained by Rádl et al. [194], 4-[(4-methoxyphenyl)amino]-1,3-dimethyl-1*H*-pyrazolo[3,4-*b*]quinoline (Figure 22, R = OMe), was included in the modeling procedure for the binding site identification and the docking study of human β-arrestin by Chintha et al. [209].

**Figure 23:**
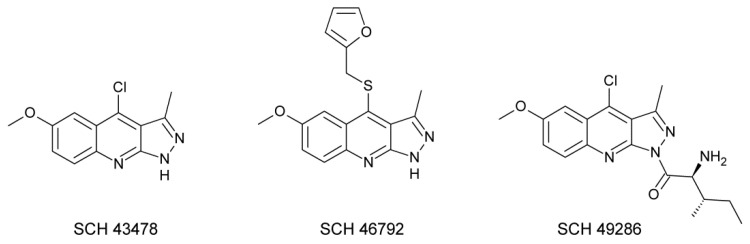
4-chloro-3-methyl-6-methoxy-1*H*-pyrazolo[3,4-*b*]quinoline (SCH 43478); 4-[[(furan-2-yl)methyl]sulfanyl]-3-methyl-6-methoxy-1*H*-pyrazolo[3,4-*b*]quinoline (SCH 46792); and (2*S*,3*S*)-2-amino-1-(4-chloro-6-methoxy-3-methyl-1*H*-pyrazolo[3,4-*b*]quinolin-1-yl)-3-methylpentan-1-one (SCH 49286).

Unfortunately, the results for that compound were not amazing. Another derivative, 4-[(4-ethoxyphenyl)amino]-1,3-dimethyl-1*H*-pyrazolo[3,4-*b*]quinoline (Figure 22, R = OEt), was used by Vyas et al. [210] as one of the known potential apoptosis inducers that are used to generate pharmacophore models with their apoptosis-inducing activity, for the same reason that Kemnitzer et al. used 4-[(4-propionylphenyl)amino]-1,3-dimethyl-1*H*-pyrazolo[3,4-*b*]quinoline (Figure 22, R = COC_2_H_5_) as the reference in apoptosis-inducer studies [211]. Different pyrazoloquinoline derivatives have attracted attention as antiviral compounds against the herpex simplex virus. Albin et al. tested the activity of three earlier synthesized [200,212] derivatives (Figure 23) against herpex simplex virus type 2 (HSV-2) [213].

**Figure 24:**
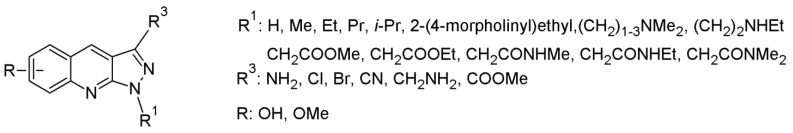
1*H*-Pyrazolo[3,4-*b*]quinoline derivatives examined by Bell and Ackerman in [214].

In vitro tests were performed with African green monkey kidney (Vero) cells, human diploid lung (WI-38) cells, human epithelial (HeLa) cells and human foreskin fibroblast (FS-85) cells. The monolayers of the cells were infected with HSV-2 in the presence or absence of the compounds SCH 43478, 46792 and 49286. The results were compared with those that were obtained with Acyclovir (ACV), which is the most popular drug that is used against herpes simplex. The inhibition of the HSV-2 plaque formation in Vero cells was achieved with concentrations lower than those for ACV; however, for the other cell lines, ACV is more effective. The cytotoxicity of all of the studied compounds against the tested cell lines was comparable to the effect of ACV. It is important to say that the compounds of interest were effective only when added shortly after infection; if added after 3 h or later, they were ineffective, which is a much worse result than that obtained for the ACV. Another broad group of 3-amino-1*H*-pyrazolo[3,4-*b*]quinolines (Figure 24) were studied by Bell and Ackerman [214].

**Figure 25:**
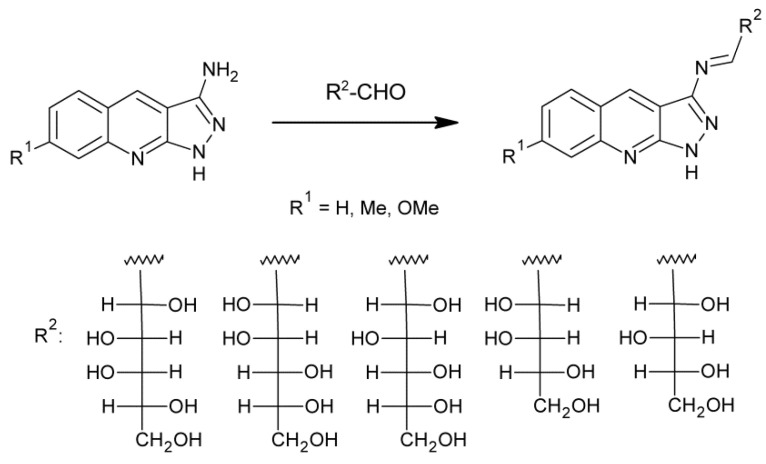
1*H*-Pyrazolo[3,4-*b*]quinoline derivatives examined by Bekhit et al. [215].

The authors tested the synthesised compounds against HSV-2 in vitro on mouse emryo fibroblast monolayers. Some of the studied compounds exhibited high activity (comparable or higher than that of Acyclovir), but they were never tested again. A different group of researchers synthesised 3-amino-1*H*-pyrazolo[3,4-*b*]quinoline that was derivatived with monosaccharides at the amino group (Figure 25), and they tested those against herpes simplex virus type 1 (HSV-1) [215].

African green monkey kidney (Vero) cells were infected with HSV-1. From the tested group, most of the compounds did not show significant cytotoxicity at the concentrtations that are safe for living cells. Only two derivatives were borderline cytotoxic: 7-methyl- and 7-methoxy-3-amino-1*H*-pyrazolo[3,4-*b*]quinoline that was derivatised with pentoses. The authors from the same research group also tested another group of derivatives, and they found that simple 7-methoxy-3-amino-1*H*-pyrazolo[3,4-*b*]quinoline has an in vitro anti-HSV-1 activity that is comparable to Acyclovir [216]. The authors also show that the compound of interest has low toxicity (tested in vivo in male mice), even when administrated through the parenteral route (nontoxic up to 80 mg/kg). The compounds that were obtained by Bekhit et al. [215] (Figure 25) were tested by Arif et al. for their potential cytotoxic activity [217,218]. At a 100 μM concentration, some of the compounds showed high cytotoxicity against human breast carcinoma cell lines (MCF-7 and MDA-MB-231); however, at the same time, they were comparably cytotoxic to normal human breast epithelial cell lines (MCF-10A and MCF-12A). The 7-Methoxy-3-amino-1*H*-pyrazolo[3,4-*b*]quinoline antiviral activity against HIV Type 1 was tested together with other different heterocyclic compounds [219]. Unfortunately, the compound did not show significant activity against that type of virus.

### 4.4. Antibacterial Activity

**Figure 26:**
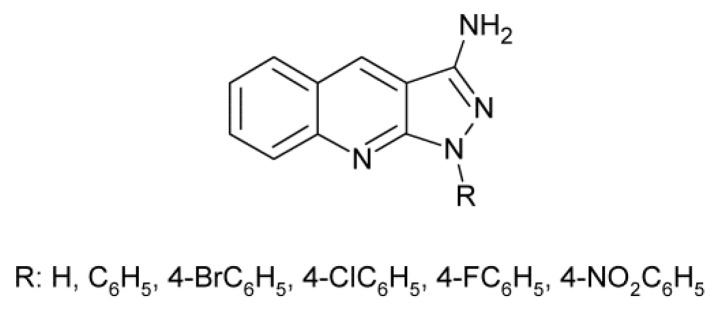
1-Substituted-3-amino-1*H*-pyrazolo[3,4-*b*]quinolines.

Because of the rising bacterial resistance to antibiotics [220], there is a constant need to invent new drugs. Pyrazoloquinoline derivatives have been also tested in this field. A group of pyrazoloquinoline derivatives was tested by El-Sayed and Aboul-Enein [221]. The authors started with 3-amino-1*H*-pyrazolo[3,4-*b*]quinoline and they substituted it at the amino group (Figure 26).

**Figure 27:**
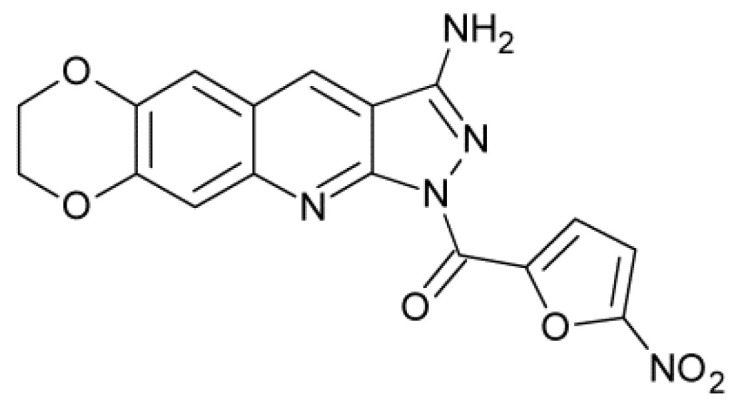
3-amino-1-(5-nitro-2-furoyl)-6,7-ethylenedioxy-1*H*-pyrazolo[3,4-*b*]quinoline.

The compounds were then tested against *E. coli*, *S. aureus*, *C. albicans* and *A. niger*, and they were compared with ampicilin and ketoconazole. The preliminary results indicated that none of the tested compounds had activity higher than ampicilin against *E. coli*. 1-(4-fluorophenyl)- and 1-(4-nitrophenyl)-3-amino-1*H*-pyrazolo[3,4-*b*]quinoline achieved results that were comparable to ampicilin against *S. aureus*. Both compounds also have high activity against *C. albicans* and *A. niger*, with the second of them even higher than ketoconazole. In the case of *A. niger*, 1-(4-chlorophenyl)-3-amino-1*H*-pyrazolo[3,4-*b*]quinoline also showed high activity. Another family of differently substituted 3-amino-1*H*-pyrazolo-[3,4-*b*]quinolines was synthesised by Lapa et al. [222]. Among the group, one compound, (Figure 27) exhibited in vitro activity against *S. aureus, S. epidermidis* and *S. pneumonia* that was comparable to Kanamycin (MIC = 4.0–8.0 μg/mL).

**Figure 28:**
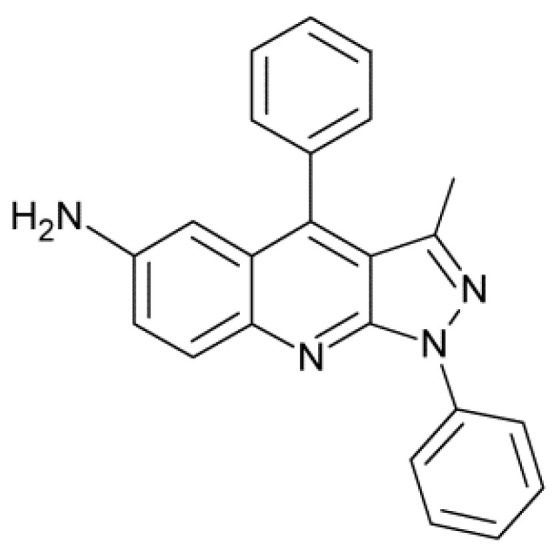
6-amino-3-methyl-1,4-diphenyl-1*H*-pyrazolo[3,4-*b*]quinoline.

Against *Mycobacterium smegmatis*, the compound was even more active than Kanamycin. A different amino derivative was tested by Hamama et al. [54]. The authors used the Path 3 synthetic methodology (Figure 3), reacted arylidene compound **74** with 1,4-phenylenediamine and obtained 6-amino-3-methyl-1,4-diphenyl-1*H*-pyrazolo-[3,4-*b*]quinoline (Figure 28).

**Figure 29:**
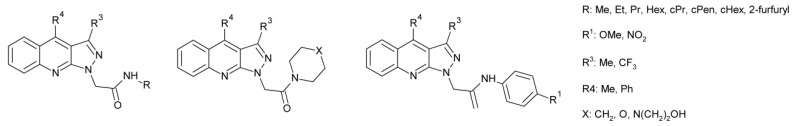
1*H*-Pyrazolo[3,4-*b*]quinoline, synthesised and tested by Jitender et al. [83].

The in vitro activity of the compound against *Bacillus subtilis* and *Escherichia coli* was a little lower than that found for Ampicilin. Pyrazoloquinolines of a totally different structure were studied by Quiroga et al. [118] (Scheme 45). The authors obtained a series of benzo[*h*]pyrazolo[3,4-*b*]quinolin-5,6-diones (**165**), between which 3-methyl-1,4-diphenyl-, 3-methyl-4-(4-methylphenyl)-1-phenyl- and 3-methyl-4-(4-fluorophenyl)-1-phenyl- seemed to be the most promising and presented the strongest in vitro activity against some *Mycobacterium* spp. For two active compounds, the authors also presented the XRD measurement results. The results indicate that the research should be continued. Another group of pyrazoloquinoline derivatives that were differently substituted at N-1 (Figure 29) were synthesised and tested by Jitender et al. [83].

**Figure 30:**
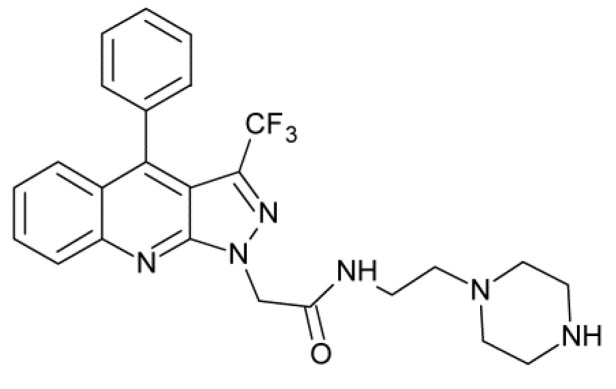
2-(4-phenyl-3-(trifluoromethyl)-1*H*-pyrazolo[3,4-*b*]quinolin-1-yl)-*N*-(2-(piperazin-1-yl) ethyl) acetamide.

A group of 32 compounds was tested for antibacterial activity against different Gram-positive (*S. aureus, B. subtilis, M. luteus*) and Gram-negative (*E. coli, K. planticola, P. aeruginosa*) bacteria. From the group, only four compounds had some activity, and the most potent was 2-(4-phenyl-3-(trifluoromethyl)-1*H*-pyrazolo[3,4-*b*]quinolin-1-yl)-*N*-(2-(piperazin-1-yl)ethyl) acetamide (Figure 30), which, with an MIC at 3.9–7.8 μg/mL, was a little worse than the value that was found for Ciprofloxacin (the reference in the study).

Better results were obtained in the activity against *Candida albicans* spp.; for all the tested species, 2-(4-phenyl-3-(trifluoromethyl)-1*H*-pyrazolo[3,4-*b*]quinolin-1-yl)-*N*-(2-(piperazin-1-yl)ethyl) acetamide was as active as the Miconazole biofilm inhibition assay of the studied compound, which was comparable to the results for Ciprofloxacin against Gram-positive and Gram-negative bacteria, and to the results for Miconazole against *C. albicans*. All of the 32 compounds were also tested for their cytotoxicity against the HeLa, HepG2, A549 and COLO 205 cancer cells lines, but the IC_50_ (μM) values had to be at least one level of magnitude higher than those found for 5-fluorouracil (as a reference) in order to attract more attention as an anticancer drug.

### 4.5. Anticancer Activity

**Figure 31:**
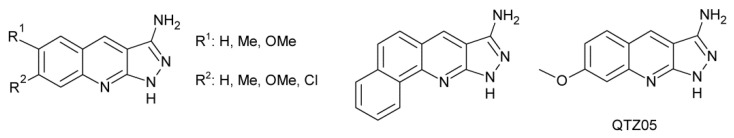
3-amino-1*H*-pyrazolo[3,4-*b*]quinoline derivatives tested by Karthikeyan et al. [225].

Cancer is a leading cause of death (after cardiovascular diseases), and, in 2020, there were nearly 10 million deaths that were attributed to it (WHO). At the moment, world medicine has many anticancer drugs, but most of them are also cytotoxic to normal cells, and especially to those that are rapidly dividing. Thus, there is still a need for new highly selective anticancer drugs [223,224]. 7-Methoxy-3-amino-1*H*-pyrazolo[3,4-*b*]quinoline, which appeared to be as active as Acyclovir against HSV-1 [216], was also tested as an inhibitor of the growth of cancer cells. Karthikeyan et al. tested the cytotoxicity of a group of 3-amino-1*H*-pyrazolo[3,4-*b*]quinolines (Figure 31) against ten cancer cell lines, including breast, colon, prostate, brain and ovarian, in comparison to a noncancerous cell line, the human embryonic kidney [225].

**Figure 32:**
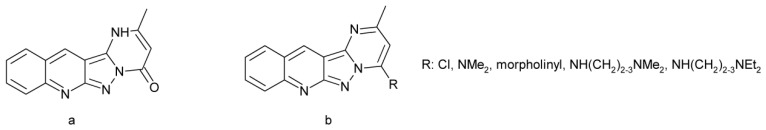
The structures of: (**a**) 2-methylpyrimido[1″,2″:1,5]pyrazolo[3,4-*b*]quinoline-4(1*H*)-one; and (**b**) 4-R-2-methylpyrimido[1″,2″:1,5]pyrazolo[3,4-*b*]quinolines.

The cytotoxicity was determined by MTT assay by using different concentrations for every compound, in the range of 0.1–100 μM. The most potent in the series turned out to be 7-methoxy-3-amino-1*H*-pyrazolo[3,4-*b*]quinoline (QTZ05), which was effective against all of the tested colon cancer cell lines (HCT-116, HCT-15, HT-29 and LOVO) and the brain cancer cell line (LN-229) at a concentration of 10.2 μM or lower. As can be seen (Figure 2 and Figure 4 in [225]), the compound of interest decreased the density and the colony size. Additionally, as the authors point out, the HCT116 cells that survived were unable to replicate. The results indicate that 7-methoxy-3-amino-1*H*-pyrazolo[3,4-*b*]quinoline produces an increase in the number of cells in the sub G1 phase of the cell cycle. The authors also checked the ability of the studied compound to induce apoptosis. The same authors (Karthikeyan et al.) also studied a different group of 1*H*-pyrazolo[3,4-*b*]quinoline derivatives (Figure 32) in the search for an anticancer drug [79,226].

**Figure 33:**
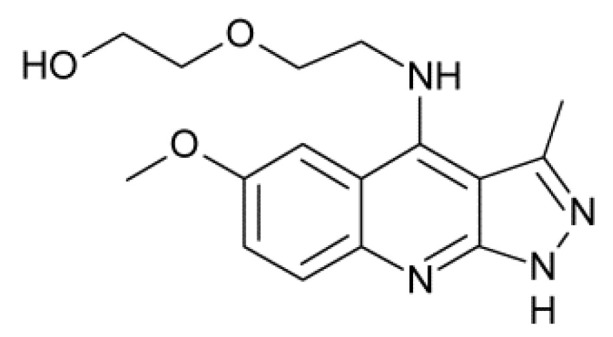
The structure 6-methoxy-4-[2-[(2-hydroxyethoxyl)ethyl]amino]-3-methyl-1*H*-pyrazolo-[3,4-*b*]quinoline (SCH 51344).

2-Methylpyrimido[1″,2″:1,5]pyrazolo[3,4-*b*]quinoline-4(1*H*)-one (Figure 32a) appeared to be the most active against colon cancer cells (HCT-116 and S1) and prostate cancer cells (PC3 and DU-145) at concentrations of 0.6–1.2 μM. At the same time, it was 10–15 times less cytotoxic to normal cells (canine kidney MDCK, mouse fibroblasts NIH/3T3 and human embryonic kidney HEK293/pcDNA.3.1.). 2-Methylpyrimido-[1″,2″:1,5]pyrazolo[3,4-*b*]quinoline-4(1*H*)-one was also found to be the most active in reversing the ABCG-2-mediated resistance to mitoxantrone, doxorubicin and cisplatin, which are common anticancer drugs. Mutations in Ras proteins are able to lead to unregulated cell division and are found in a significant number of human cancers [227]. For this reason, the identification of drugs that inhibit, either directly or indirectly, the transforming activity of the Ras protein is important in the search for an effective treatment of cancer [228]. 6-Methoxy-4-[2-[(2-hydroxyethoxyl)ethyl]amino]-3-methyl-1*H*-pyrazolo[3,4-*b*]quinoline (SCH 51344, Figure 33) was one of the compounds that was tested as a possible *ras*-transformation inhibitor.

**Figure 34:**
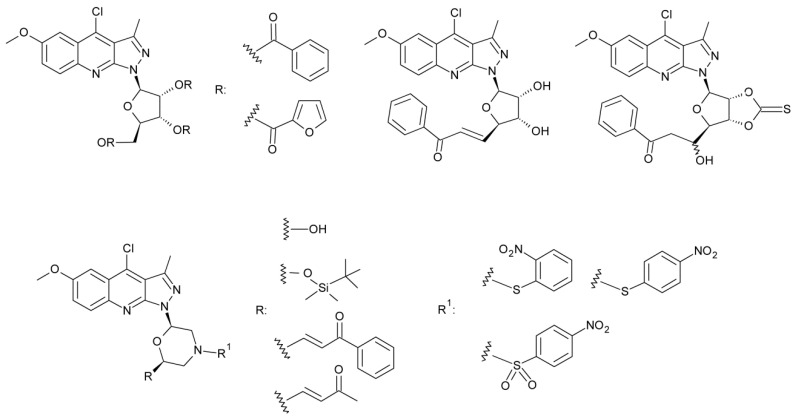
Selected (most effective) 1-substituted-4-chloro-3-methyl-6-methoxy-1*H*-pyrazolo[3,4-*b*]quinoline derivatives.

Kumar et al. carefully studied the mechanism of inhibition [229,230,231,232]. Pyrazoloquinoline SCH 51344, specifically, inhibits the RAS-mediated cell morphology pathway (H-, K- and N-RAS V12-induced membrane ruffling). The treatment of RAS-transformed cells with SCH 51344 restored organised actin filament bundles. Moreover, the anchorage-independent growth of K-RAS, which transformed NIH 3T3 cells and human colon and pancreatic tumor-derived cells (DLD-1, Panc-1, SW-480), was inhibited by SCH 51344. The authors also show that SCH 51344 is not cytotoxic to normal cells. Their studies show that the compound selectively suppressed oncogene-transformed growth without the gross effect on the normal signalling pathway. Gelman et al. also found that SCH 51344 suppresses *src*-, *ras*- and *raf*-induced oncogenic transformation by more than 90% at a 40 μM concentration of the pyrazoloquinoline [233]. The oncogenic growth potentials of rat-6/*ras* and /*raf* are more sensitive to SCH 51344 than rat-6/*src* cells because they are more dependent on pathways that are blocked by the compound. (*S*)-Crizotinib was tested as an anticancer drug by Huber et al., and the obtained results were compared to (*R*) enantiomer and SCH 51344 [234,235]. The authors, by using the proteomic approach, identified the target of SCH 51344 as the human mutT homologue MTH1 (NUDT1). The analysis of that pyrazoloquinoline was only one step to the main goal, which was the study concerning (*S*)-crizotinib. SCH 51344 was also one of the compounds that was tested by Ursu and Waldman as a small molecule target that binds to MTH1, with a comparison to (*R*)- and (*S*)-crizotinib [236]. The authors tested a few molecules of different structures by linker-based target identification techniques. The authors attached SCH 51344 to sepharose and proved that MTH1 is a primary target of the compound. The result was also confirmed in vitro by isothermal titration calorimetry and an MTH1 catalytic assay. SCH 51344 (6-methoxy-4-[2-[(2-hydroxyethoxyl)ethyl]amino]-3-methyl-1*H*-pyrazolo[3,4-*b*]quinoline) is now commercially available as an MTH1 inhibitor [237]. It is in use as one of the reference compounds in research that concerns anticancer drugs [238,239,240,241,242,243]. Different pyrazoloquinoline derivatives, functionalized with ribose or morpholine moieties (Figure 34), have been also tested as antitumor agents [244,245,246].

**Figure 35:**
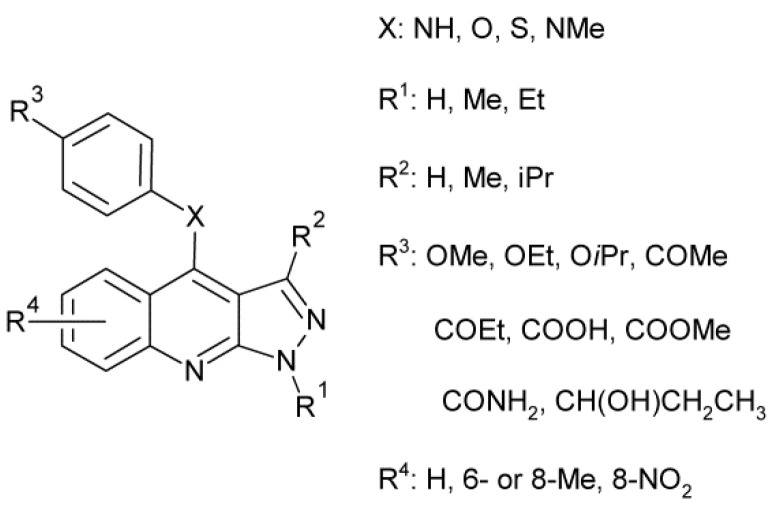
Pyrazolo[3,4-*b*]quinolines tested by Zhang et al. [57].

The mechanism of inhibition is probably the binding to an allosteric region of the *Ras* p21 protein, which leads to conformational change and prevents the binding of ^3^H-GDP to the protein.The best results have been obtained for 1-[4-chloro-3-methyl-6-methoxy-1*H*-pyrazolo[3,4-*b*]quinolinyl]-1-riboburanoside-tribenzoate,-tris(2-furoate),-4-(*E*-2-benzoylethenyl),4-[(1-hydroxy-2-benzoyl)ethyl]-2,3-thiocarbonate: a >70% inhibition at 10, 10.5, 10 and 1.5 μM concentrations, respectively. For the morpholine series, eight derivatives showed >70% inhibition at a <10μM concentration. One of the ways of anticancer drug action is by inducing the apoptosis of tumor cells [247]. 4-Phenylamino pyrazolo[3,4-*b*]quinolines, which have been tested for their antiviral activity [194,198], have also brought attention to themselves as potential apoptosis inducers. Zhang et al. tested a group of *N*-phenyl-1*H*-pyrazolo-[3,4-*b*]quinolin-4-amines (Figure 35) as potent apoptosis inducers [57].

The authors tested the activity of the synthesised derivatives against the human breast cancer (T47D), colon cancer (HCT116) and liver cancer (SNU 398) cell lines in vitro. The most active against the chosen cancer cells appeared to be 1,3-dimethyl-*N*-(4-propionylphenyl)-1*H*-pyrazolo[3,4-*b*]quinolin-4-amine (Figure 35, X = NH, R^1^,R^2^ = Me, R^3^ = COEt, R^4^ = H), which was about 6 ÷ 13-fold more potent than the reference, *N*-(4-acetylphenyl)-2,3-dihydro-1*H*-cyclopenta[*b*]quinolin-9-amine. The authors also tested the cell proliferation activity of the same compound and they again obtained very promising results; however, the research has not been continued.

### 4.6. Antiparasitic Activity

Al-Qahtani et al. tested the effect of the series of pyrazoloquinoline derivatives that were obtained earlier by Bekhit et al. [215] (Figure 25) on the growth of *Leishmania donovani* protozoan parasites [248]. The *L. donovani* (strain DD8) were cultures in Schneider’s medium with four different concentrations of drugs (50–400 μM), and they were compared to Amphotericin B-treated cells and to an untreated control. The results indicate that some of the derivatives exhibited an activity that was comparable to the classic drug Amphotericin B; however, the effect was observed after 12 h compared to 6 h for Amph. B. The research has been never repeated.

### 4.7. Treatment of Schizophrenia

**Figure 36:**
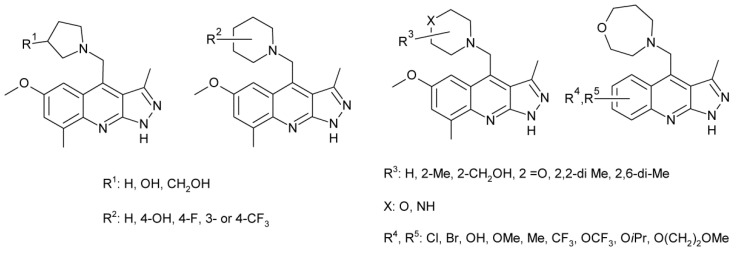
1*H*-Pyrazolo[3,4-*b*]quinolines synthesized and tested by Yang et al. and Ho et al. [91,92].

As schizophrenia is affecting circa 1% of the world’s population [249], and because the already existing drugs enable only a small portion of patients to lead independent lives, there is still the need for improved treatment of schizophrenia. Antipsychotic drugs, which are used in the treatment of schizophrenia, are mostly dopamine 2 (D2) receptor antagonists [250]. PDE10, which is a dual cAMP/cGMP phosphodiesteraze, and which belongs to a family of degradative enzymes that hydrolyse the second messengers that terminate signal transduction, is expressed at high levels in the striatal medium spiny neurons. The inhibition of PDE10, and the following blocking of the degradation of cGMP and cAMP, should mimic the effect of a D2 receptor antagonist and a D1 receptor agonist, which is an ideal profile for an antipsychotic drug. A wide family of different pyrazoloquinoline derivatives was tested in the search for potent PDE10 inhibitors for the treatment of schizophrenia. In the first group, which was analyzed by Yang et al. and Ho et al. [91,92], 3-methyl-1*H*-pyrazolo[3,4-*b*]quinolines could be found, which were substituted at position 4 with different saturated heterocyclic rings (Figure 36).

**Figure 37:**
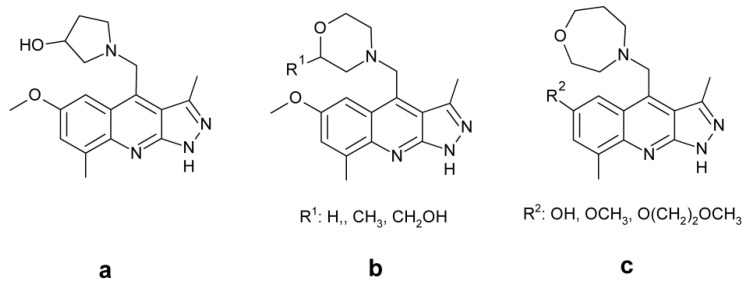
(**a**) *N*-[6-Methoxy-3,8-dimethyl-1*H*-pyrazolo[3,4-*b*]quinolinyl]methyl-3-hydroxypyrollidine; (**b**) *N*-[6-methoxy-3,8-dimethyl-1*H*-pyrazolo[3,4-*b*]quinolinyl]methyl-2-R^1^-morpholines; (**c**) *N*-[6-R^2^-8-methyl-1*H*-pyrazolo[3,4-*b*]quinolinyl]methyl-1,4-oxazepanes.

The synthesised compounds were tested for their affinity to the cloned human recombinant, PDE10A1, by measuring their ability to compete with [^3^H]cAMP. The strongest binding affinities were obtained for one pyrrolidine derivative (Figure 37a), three morpholine-derivatized compounds (Figure 37b) and three 1,4-oxazepane-derivatives (Figure 37c) with *K*_i_ 0.6–5 nM.

**Figure 38:**
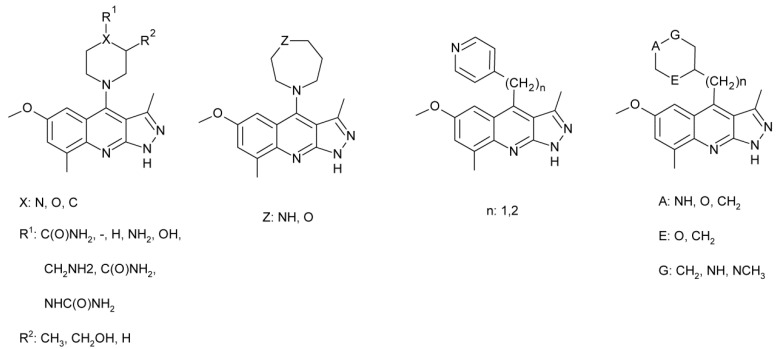
1*H*-Pyrazolo[3,4-*b*]quinolines synthesized and tested by Ho et al. [92].

One of the following, *N*-[6-methoxy-3,8-dimethyl-1*H*-pyrazolo[3,4-*b*]quinolinyl]-methylmorpholine, was used for co-crystallisation with PDE10A, and the obtained crystals were studied with XRD. The second group was obtained by Ho et al. [92] and it is presented in Figure 38.

**Figure 39:**
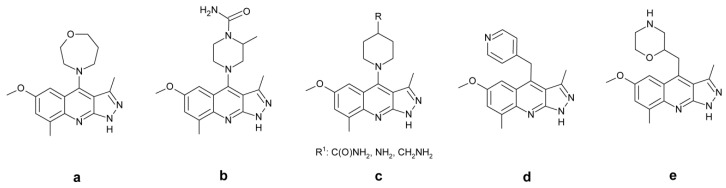
(**a**) 4-[6-Methoxy-3,8-dimethyl-1*H*-pyrazolo[3,4-*b*]quinolinyl]-1,4-oxazepane; (**b**) 1-[6-methoxy-3,8-dimethyl-1*H*-pyrazolo[3,4-*b*]quinolinyl]-4-carboxamid-3-methylpiperazine; (**c**) 1-[6-methoxy-3,8-dimethyl-1*H*-pyrazolo[3,4-*b*]-quinolinyl]-4-R-piperidines; (**d**) 4-[6-methoxy-3,8-dimethyl-1*H*-pyrazolo[3,4-*b*]quinolinyl]methylpyridine; and (**e**) 2-[6-methoxy-3,8-dimethyl-1*H*-pyrazolo[3,4-*b*]quinolinyl]methylmorpholine.

The strongest binding affinities were obtained for one 1,4-oxazepane-derivative derivative (Figure 39a), one piperazine-derivative (Figure 39b), three piperidine-derivatized compounds (Figure 39c), one pyridyne-derivative (Figure 39d) and one morpholine-derivatised compound (Figure 39e) with *K*_i_ 0.6–5 nM.

**Figure 40:**
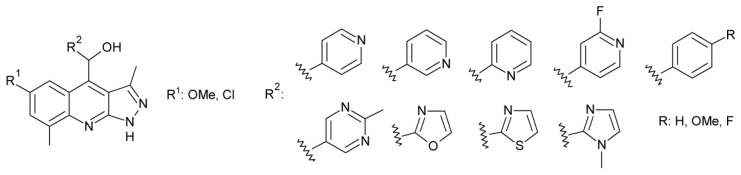
1*H*-Pyrazolo[3,4-*b*]quinolines synthesised and tested by McElroy et al. [93].

One of the tested piperazine derivatives had been co-crystallised with PDE10A. Both author groups (Yang et al. and Ho et al.) state that they had been planning to perform in vivo evaluations; however, we could not find any further results concerning the pyrazoloquinolines described above. In the different families of pyrazoloquinoline derivatives, the moiety in position 4 was secondary alcohol with an aromatic moiety (Figure 40) or some other substituents (they are not presented here because of worse results).

The most potent in in vitro binding to PDE10A appeared to be 4-[(4-pyridyl)hydroxymethyl]- and 4-[[4-(3-fluoropyridyl)] hydroxymethyl]-6-methoxy-3,8-dimethyl-1*H*-pyrazolo[3,4-*b*]quinoline, with a *K*i equal to 0.06–0.07 nM. The first of them was explored extensively by Wu et al. by 3D-QSAR, molecular docking and molecular dynamics simulations [251], together with other derivatives that were obtained by McElroy et al. [93]. Three of the derivatives: 4-[(4-pyridyl)hydroxymethyl]-6-methoxy-3,8-dimethyl-1*H*-pyrazolo[3,4-*b*]quinoline, 1-[6-methoxy-3,8-dimethyl-1*H*-pyrazolo-[3,4-*b*]quinolinyl]methyl-4-fluoropiperidine and 1-[6-methoxy-3,8-dimethyl-1*H*-pyrazolo[3,4-*b*]-quinolinyl]-4-methoxypiperidine, which were studied earlier [91,92,93], have been used as the reference compounds in the search for new PDE10A inhibitors on the basis of the quinazoline derivatives [252].

### 4.8. Treatment of Diabetes

**Figure 41:**
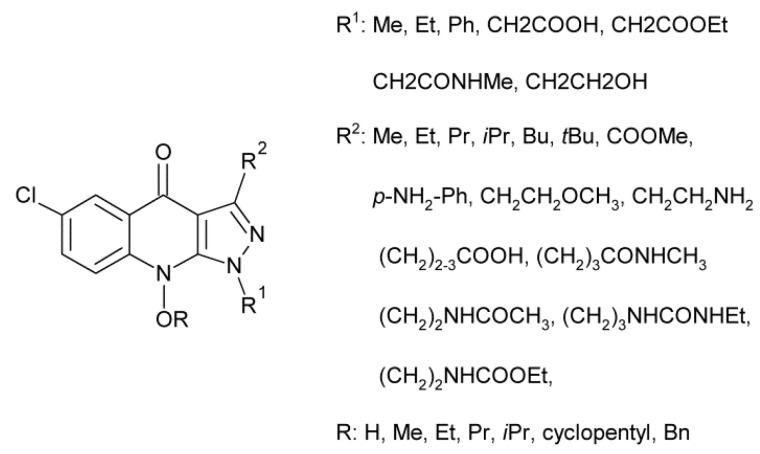
The structure of 1,9-dihydro-9-hydroxypyrazolo[3,4-*b*]quinolin-4-ones derivatives.

Type 2 diabetes is now one of the most prevalent metabolic diseases worldwide [253]. Patients with type 2 diabetes are insulin resistant, and so there is still a need to find drugs that can help to increase insulin sensitivity. C-Jun N-terminal kinase-1 (JNK1) is one of the mitogen-activated protein kinases that probably plays a key role in linking insulin resistance and obesity [254]. Liu et al. synthesised a group of 1,9-dihydro-9-hydroxy pyrazolo[3,4-*b*]quinolin-4-ones derivatives (Figure 41) and tested themas JNK inhibitors [255].

**Figure 42:**
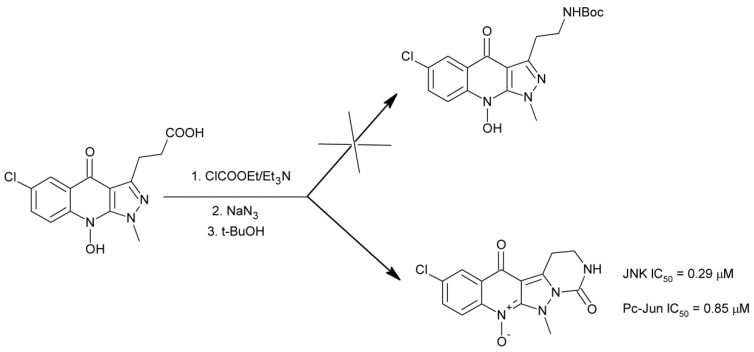
The most potent unexpected product of acid derivatisation.

All of the obtained compounds were tested in a JNK1 enzymatic inhibition assay and in a cell-based assay by measuring the inhibition of the TNFα (tumor necrosis factor)-stimulated phosphorylation of c-Jun in HepG2 (human liver cancer) cells. The most potent (JNK1 IC_50_ < 1 μM) appeared to be derivatives with R = H, R^2^ = Me and R^1^ = Et, and Bu, (CH_2_)_3_CONHMe, (CH_2_)_2_NHCOMe, (CH_2_)_3_NHCONHEt or (CH_2_)_2_NHCOOEt. The authors were also able to grow the co-crystals of one of the derivatives (R = H, R^1^, R^2^ = Me, JNK1 IC_50_ = 1.22 μM), bound into the ATP site of JNK1, and they analysed the interactions between the protein and the inhibitor. The most potent against JNK1 and also Pc-Jun proved to be the compound that was serendipitously found by the authors who tried to convert carboxylic acid to amine (Figure 42).

### 4.9. Benzodiazepine Receptor Inhibitor

**Figure 43:**
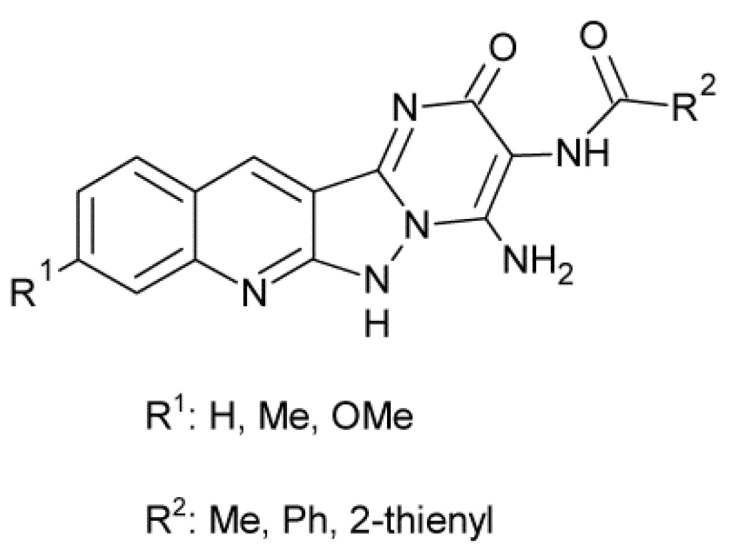
1*H*-Pyrazolo[3,4-*b*]quinolines, synthesised and tested by El-Sayed et al. [256].

The group of pyrazoloquinolines that were substituted by fusing the pyrazol ring with a purine moiety were synthesised by the funcionalisation of 3-amino-1*H*-pyrazolo-[3,4-*b*]quinoline derivatives by El-Sayed et al. (Figure 43) [256].

**Figure 44:**
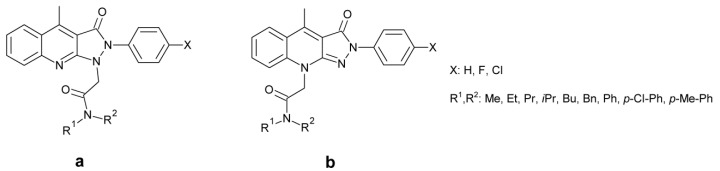
Pyrazoloquinoline derivatives obtained by Cappelli et al. [257].

The authors tested the benzodiazepine receptor (BZR) binding affinity of nine different derivatives, and they observed the best results for 2-thienyl substituent at the 2 position: an 83% inhibition of specific [3H]Ro15-1788 (Flumazenil) binding at a 10 μM concentration was achieved for 2-(2-thienyl)quinolino[2′,3′-5,4](3-pyrazolino)[3,2-*b*]purin-4-one (R1 = H, R2 = 2-thienyl). It is important to say that the *K*i value for the tested compound is three levels of magnitude higher than that found for Flumazenil. The authors have not continued their research. A different group of pyrazoloquinoline derivatives (Figure 44) were tested by Cappelli et al. as translocator protein (TSPO) ligands [257].

TSPO is an alternative binding site of diazepam [258]; however, what is more important is that a dramatic increase in the TSPO density occurs in glial cells in response to brain inflammation or injury. Many neuropathological conditions (e.g., Alzheimer’s disease, Parkinson’s disease) also increase the TSPO density [259]. The authors decided to synthesise the pyrazoloquinolines that are shown in Figure 44 because they are structurally similar to alpidem, which is a known drug that is used for the treatment of anxiety [260]. The authors performed in vitro binding experiments to rat cerebral cortex membranes together with in vivo light/dark box tests in mice (for the most promising derivatives). Both experiments showed that the highest TSPO binding affinities were achieved in vitro by *N*-R2-2-[2-(4-X-phenyl)-4-methyl-3-oxo-2*H*-pyrazolo[3,4-*b*]quinolin-9(3H)-yl]-*N*-R1-acetamides (Figure 44b). The IC_50_ values for all of these derivatives were lower than 1 nM. Moreover, the antianxiety activity of one of the derivatives (*N*-butyl-2-[2-phenyl-4-methyl-3-oxo-2*H*-pyrazolo[3,4-*b*]quinolin-9(3H)-yl]-*N*-methylacetamide) was high, at 10 mg/kg dose, which was comparable to the value that was found for emapunil with the same dose, and for diazepam with a 1 mg/kg dose. The biological activity studies were combined with a detailed structural analysis, including XRD and computational calculations. The obtained results indicate that these group of pyrazoloquinoline derivatives could be a group of new TSPO modulators, and that they are worth further research.

### 4.10. Singlet-Oxygen-Generating Activity for SPA

A scintillation proximity assay (SPA) is a biochemical screening radio-isotopic method that is used for the sensitive and rapid measurement of a broad range of biological processes [261]. Pai et al. used 4-[(4-aminophenyl)sulfanyl]-6-methoxy-3-methyl-1*H*-pyrazolo[3,4-*b*]quinoline (SCH 46891), which was obtained earlier by Afonso et al. [212], as one of the tested potent inhibitors of the phosphopeptide binding to the growth factor receptor-bound protein 2 (*Grb2*) SH2 domain [262]. The studies reveal that the inhibition of the *Grb2* SPA by SCH 46891 was light-dependent. The compound was inactive when the assay plates were kept dark. The same light-dependent inhibition property was found for the tyrosine-protein kinase (*Syk*) SH2 domain via SPA. The authors concluded that the light-dependent inhibition mechanism is based on the singlet-oxygen-generating property of SCH 46891 upon irradiation (positive reaction with singlet oxygen trap 2-methylfuran, and the suppression of the inhibition activity by singlet oxygen quenchers).

### 4.11. Pregnancy Interceptive

In 1991, Mehrotra et al. [263] tested 3-amino-6,7-dimethoxy-1*H*-pyrazolo-[3,4-*b*]quinoline as a potential pregnancy interceptor in vivo in hamsters and guinea pigs. When the compound was used in the early postimplantation schedule, it interrupted the pregnancy partially; however, it was ineffective in the preimplantation schedule. The same compound was tested in vitro on growing trophoblasts, and it appeared to prevent growth and to cause the degeneration of the cells. The results led the authors to the conclusion that the compound intercepts pregnancy probably through a direct effect on the embryo attachment site. The research was never continued or repeated with that, nor with any other pyrazoloquinoline derivative.

## 5. Conclusions

In summary, the main aim of this review was to introduce readers to the methods and procedures that are used for the 1*H*-pyrazolo[3,4-*b*]quinoline skeleton synthesis, and to familiarize them with some of the photophysical and biological properties. The work is based on publications that appeared in the years 1911–2021. During this period, approx. 350 publications and patents were published, and, to the best of our knowledge, this is the first study of this type. We wanted to collect, in this review, all of the most important synthetic methods to create a kind of guide for researchers who are involved in the synthesis of nitrogen-condensed heterocycles, and who may decide to direct their interests there.

By studying the development of the synthetic methods of 1*H*-pyrazolo[3,4-*b*]quinolines over the last one hundred years, a significant revolution in the approach to preparative methods can be noticed, and especially in the last ten years. In the initial period, syntheses based on classical reactions (e.g., the Friedländer condensation or modifications of the Niementowski reaction) were used. Nowadays, an increasing number of publications can be found that describe three-/four-component reactions, and reactions that are carried out in an aqueous environment with the use of a microwave field, or with ultrasounds and ionic liquids, which are catalysed with palladium compounds and nanoparticles, or that are even mediated with baker’s yeast. All of these procedures are part of the so-called “green chemistry”.

These methods lead to the synthesis of the basic skeleton, as well as functionalized systems, which offer further possibilities for structural modifications with hydroxyl, halogen or amino moieties. The latter aspect is especially important for chemists in the pharmaceutical industry.

The first synthesised 1*H*-pyrazolo[3,4-*b*]quinolines attracted the attention of researchers with their emissive properties, and they were applied as optical brighteners in the 1960s. Later studies show that, in some cases, the quantum yield of the fluorescence was equal to one, and that, in addition, these compounds turned out to be very resistant to temperature or oxidation. For this reason, they were used as emission materials for the fabrication of organic light-emitting diodes (OLEDs) that work on the basis of the phenomenon of fluorescence. In recent years, however, very efficient emissive materials that are based on the phenomenon of phosphorescence or thermally activated delayed fluorescence TADF have appeared, and so it should not be expected that pyrazoloquinolines will be used in the future to fabricate OLEDs. However, by taking into account their very good emissive properties and their stability, it can be expected that they will be used as fluorescent sensors for various analytes, as dyes for synthetic fibers, as dyes for fluorescence microscopy or as materials for the security features of banknotes based on fluorescence.

The third aspect of the use of pyrazoloquinolines are their biological properties, which have been studied since the 1970s, and which were started by Siminoff and Crenshaw, who investigated the antimalarial properties of these compounds. Currently, the spectrum of biological properties is much broader and includes a variety of tests, among which the research on antibacterial, antiviral and anticancer properties is at the forefront. This resulted in, among other things, the commercialization of some preparations, such as, for example, 6-methoxy-4-[2-[(2-hydroxyethoxyl)ethyl]amino]-3-methyl-1*H*-pyrazolo[3,4-*b*]quinoline, which is now commercially available as an MTH1 inhibitor. Therefore, further studies towards the investigation of novel pharmacologically active 1*H*-pyrazolo[3,4-*b*]quinolines are highly desirable.
